# Multi-omics analysis of a *Bacteroides fragilis* isolate from an ulcerative colitis patient defines genetic determinants of fitness in bile

**DOI:** 10.1101/2023.05.11.540287

**Published:** 2023-05-11

**Authors:** Aretha Fiebig, Matt Schnizlein, Selymar Pena-Rivera, Florian Trigodet, Abhishek Anil Dubey, Miette Hennessy, Anindita Basu, Sebastian Pott, Sushila Dalal, David Rubin, Mitchell L. Sogin, A. Murat Eren, Eugene B. Chang, Sean Crosson

**Affiliations:** a Department of Microbiology and Molecular Genetics, Michigan State University, East Lansing, MI, USA; b Department of Medicine, University of Chicago, Chicago, IL, USA; c Marine Biological Laboratory, Woods Hole, MA, USA; d Helmholtz Institute for Functional Marine Biodiversity, University of Oldenburg, Oldenburg, Germany.

## Abstract

*Bacteroides fragilis* comprises 1–5% of the gut microbiota in healthy individuals but can expand to >50% of the population in ulcerative colitis (UC) patients experiencing inflammation. The mechanisms underlying such microbial blooms are poorly understood, but the gut of UC patients has physicochemical features that differ from healthy patients and that likely impact microbial growth and community structure. In UC colectomy patients with surgically created ileoanal pouches, levels of the bile acid deoxycholate (DC) are reduced in the pouch. We developed a *B. fragilis* strain isolated from a UC patient with pouch inflammation (i.e. pouchitis) as a genetic model system and have used this strain to identify genes and pathways that impact fitness in DC and crude bile. Treatment of *B. fragilis* with a DC concentration congruent with a healthy human gut reduced cell growth and remodeled transcription of approximately one-quarter of all genes. DC strongly induced transcription of chaperones and select transcriptional regulators and efflux systems and downregulated protein synthesis genes. We further defined *B. fragilis* genes that impacted fitness in media containing DC or crude bile using a barcoded collection of ≈50,000 unique mutants. Genes that contribute to cell envelope functions including cardiolipin synthesis, cell surface glycosylation, and systems implicated in sodium-dependent bioenergetics were major bile acid fitness factors. The correspondence between genes that were transcriptionally regulated and genes that impacted fitness in bile was limited. Our study provides a genome-scale view of a *B. fragilis* bile response and genetic determinants of fitness in DC and crude bile.

**Importance:**

The Gram negative nanaerobe, *Bacteroides fragilis*, is a common member of the human gut microbiota that can colonize multiple niches within the host and impact human physiology through a variety of mechanisms. Identification of genes that enable *B. fragilis* to grow, and often bloom, across a range of host environments has been impeded in part by the relatively limited genetic tractability of this species. We have developed a high-throughput genetic resource for a *B. fragilis* strain isolated from a UC pouchitis patient that we have used to define genetic determinants of fitness in a bile acid with reduced abundance in UC pouches, where *B. fragilis* often blooms. This study has uncovered pathways and processes that impact *B. fragilis* fitness in bile and that may contribute to blooms during bouts of gut inflammation.

## INTRODUCTION

The human gut contains a vibrant community of fungi, protists, archaea, and bacteria. To survive and replicate in this environment, these microbial cells must adapt to complex and varying conditions including gradients in pH, O_2_, nutrients and host-derived compounds such as bile acids that vary longitudinally (i.e., from the stomach to the rectum) as well as transversely (i.e., from mucosa to lumen) (1–8). This physicochemically-dynamic environment influences how microbes interact with each other and their host, and thereby shapes the complex ecological networks of the gut. Studies aimed at defining the mechanisms by which indigenous bacteria adapt to and mitigate chemical stressors encountered in the gut are critical as we work to advance understanding of how bacteria survive disruptions in this ecosystem.

Disruptions to the gut environment as a result of infection, antimicrobial therapy, or surgery can result in blooms of opportunistic microbes (9). A surgical disruption common to patients with severe ulcerative colitis (UC) is ileal pouch anal anastomosis (IPAA), where the terminal ileum is joined to the rectum after a colectomy. Not surprisingly, IPAA reshapes the physiology of gut, influencing bile acid cycling, water absorption and mucosal physiology (10–14). The microbiome of the nascent ileal pouch adopts a colonic profile with increased numbers of anaerobes that have a shifted metabolism relative to microbes of the ileum (15–17). While this surgical procedure mitigates gut inflammation for some, approximately 50% of UC patients who undergo IPAA eventually develop an inflammatory condition of the ileal pouch, known as pouchitis, which is characterized by symptoms such as rectal bleeding and incontinence (18–20). The etiology of pouchitis remains unclear but several members of the pouch microbiota have been implicated (21–23) including Bacteroides fragilis, a Gram-negative, opportunistic bacterium that is occasionally isolated from the ileal pouch (23, 24). Due to its ability to thrive in diseased and non-diseased states, B. fragilis offers an interesting model for studying bacterial fitness and adaptability to environmental shifts encountered in the mammalian gut. Our goal was to develop a novel cultivar of *B. fragilis* isolated from a UC pouchitis patient to serve as a platform to study genetic factors associated with its fitness in the face of host-derived stressors.

Bile contains a complex mixture of detergent-like bile acids that are secreted into the digestive tract to aid in solubilization of dietary fats. These molecules are a stressor for gut microbes as they can disrupt membranes and cause damage to nucleic acids and proteins (25–28). Primary bile acids produced by the host are typically conjugated to amino acids, which promotes their solubility in an aqueous environment. However, the composition of the bile acid pool in the gut changes after secretion from the common bile duct as microbes hydrolyze conjugated amino acids and chemically modify the steroidal core of primary bile acids to yield secondary bile acids (5). The chemical state of bile acids (e.g., primary vs. secondary; conjugated vs. deconjugated) varies along the digestive tract, drives microbial fitness and shapes microbiota composition (29–34). Moreover, since microbes metabolize bile acids, fluctuations in the microbiota, such as those during gastrointestinal disease, cause shifts in the bile acid pool. For example, an inflamed UC pouch is linked to decreased abundance of secondary bile acids, which typically inhibit growth of gut microbes (22, 35). *B. fragilis* has been described as a ‘bile-tolerant’ species and selective media formulations for *B. fragilis* contain ox bile at a concentration that inhibits most enteric bacteria (36, 37).

As a common low abundance microbe that can bloom in conditions of inflammation, here we sought to identify B. fragilis genes and gene regulatory responses that affect its interactions with environmental bile acids using two complementary approaches: total RNA sequencing (RNAseq) and randomly barcoded transposon insertion sequencing (RB-TnSeq or BarSeq). We describe the complete genome sequence a *B. fragilis* cultivar from a UC pouchitis patient and its transcriptional response deoxycholate, a secondary bile acid. We further report the first barcoded transposon library in *B. fragilis*, which we used to conduct a genome-wide screen for genetic factors that determine resistance to both deoxycholate and a crude bile extract. This multi-omics investigation provides evidence that survival in the presence of bile acids involves multiple stress mitigation systems, many of which are also transcriptionally induced in the presence of deoxycholate. Reduced growth in the presence of deoxycholate correlates with highly reduced expression of protein synthesis machinery which may underlie control of *B. fragilis* populations in healthy individuals. A putative sodium translocating V-ATPase system, cardiolipin synthase, and select lipoprotein and surface polysaccharide biosynthesis enzymes were identified as critical determinants of fitness in the presence of deoxycholate.

## RESULTS

### The *B. fragilis* P207 genome

*B. fragilis* strain P207 was isolated from the J-pouch of a human pouchitis patient (24). To facilitate the development of *B. fragilis* P207 as a genetic model system, we sequenced genomic DNA using a combination of long- and short-read approaches and assembled the reads *de novo* using the repeat graph assembly algorithm of Flye (38), followed by an assembly polishing step in Pilon (39). The complete, circular P207 genome is 5.04 Mbp; no episomal sequences were present in the final assembly. Automated annotation using the prokaryotic genome annotation pipeline (PGAP) (40) predicted a total of 4,110 genes, with 6 ribosomal RNA loci, and one type IIC CRISPR system. *B. fragilis* P207 lacks sequence related to the *Bacteroides* pathogenicity island (BfPAI) (41) and thus does not contain known forms of the *bft* gene (42–44), which encodes the fragilysin toxin. *B. fragilis* P207 is therefore classified as a non-toxigenic *B. fragilis* (NTBF) strain.

### Deoxycholate treatment induces major remodeling of the *B. fragilis* transcriptome

**Figure 1:**
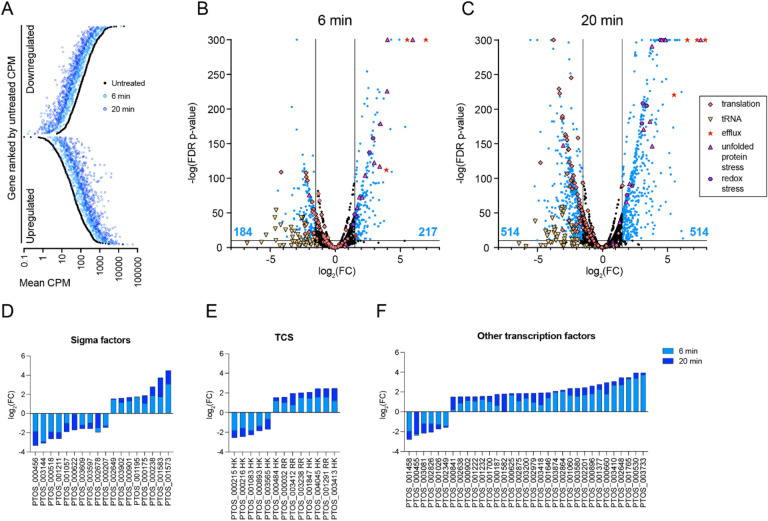
Treatment of *B. fragilis* P207 with a sub-lethal concentration of deoxycholate (DC) induces large-scale activation and repression of transcription. **(A)** Differentially transcribed genes 6 minutes (light blue circles) and 20 minutes (dark blue circles) after 0.01% (w/v) DC exposure are highly correlated. The 1028 genes that are significantly up- and downregulated by 20 minutes post DC treatment relative to untreated control (black circles) are ranked by transcript counts per million (CPM) of gene expression in the untreated condition. **(B-C)** Volcano plots of differentially transcribed genes at 6 minutes and 20 minutes post DC exposure. Lines indicate the thresholds of significance used in this study (FDR p < 10^−10^, log_2_(fold change) < −1.5 or >1.5, where fold change (FC) reflects CPM after DC exposure / CPM before DC exposure). The number of significantly up- or downregulated transcripts (blue points) at each time point is indicated at the bottom of graph (blue text). Genes that do not change significantly are in black. Functional categories of genes are highlighted with special symbols include: a) Translation processes (orange diamonds; GO terms: 0003735, 0006414, 0006400, 0043022, 0000049), b) transmembrane efflux processes (red stars; operon *PTOS_003611-3614*), c) Unfolded protein stress (pink triangles; GO term: 0051082), d) redox stress (purple hexagon; catalase, *msrB*, and *dps*, e) tRNA (also related to translation processes) are highlighted as yellow inverted triangles **(D-F)** log_2_(fold change) in transcript levels of genes encoding significantly regulated **(D)** alternative sigma factors, **(E)** two-component signaling (TCS) proteins (HK – histidine kinase, RR – response regulator), and **(F)** other transcription factors after 6 or 20 minutes of DC exposure (light and dark blue, respectively).

As a first approach to identify *B. fragilis* genes that function in adaptation to bile acid exposure, we conducted an RNA sequencing experiment to identify transcripts that have altered abundance upon treatment with the secondary bile acid, deoxycholate (DC). We compared RNA abundance from *B. fragilis* P207 exposed to 0.01% (w/v) DC for either 6 or 20 minutes to pre-treatment control sample. This concentration of DC marginally inhibited growth of *B. fragilis* P207 (Figure S1). The number of transcripts with significant changes in abundance was large, indicating that DC exposure induces a major shift in *B. fragilis* physiology. After 20 minutes, over one-quarter of the transcriptome (1028 of 4110 genes) had an absolute log_2_(fold change) > 1.5 (FDR p-value <10^−10^) (Table S1, Figure 1).

The transcriptional response of *B. fragilis* P207 to DC at the 6 min and 20 min time points was highly correlated and, as expected, absolute changes in transcript levels were higher at 20 minutes relative to the control sample (Figure 1A). Transcript levels of 217 genes were significantly higher and 184 genes were lower than the untreated control (FDR p-value < 10^−10^, |log_2_(fold change)| > 1.5) 6 min after DC exposure. After 20 minutes, an equal number of genes (514) had higher or lower transcript levels than the control (Figure 1B–C). To identify regulatory patterns in functional sets of genes, we assigned Interpro (45) and GO terms (46) to each gene in the P207 genome (Table S2). We then used gene set enrichment analysis (GSEA) (47), and Fisher’s exact test (48) to identify gene function classes that are significantly up or- downregulated after 20 minutes of DC treatment. Below, we highlight functional classes of genes that are significantly (FDR < 0.05) activated or repressed by DC exposure.

#### Multiple classes of transcription factors are regulated by DC

The massive transcriptional response that we observe upon DC treatment is likely a result of a cascade of transcriptional regulatory events cued by stress inflicted by deoxycholate. Two of the top three enriched functional categories in the activated gene set relate to transcription (enriched GO terms: transcription cisregulatory region binding, and regulation DNA-templated transcription). The *B. fragilis* sigma factors had a striking profile in which eight sigma factors were activated and ten repressed (|log_2_(FC)|>1.5) by the 20 minute time point, (Figure 1C; Tables S1 & S2). Other common classes of transcriptional regulators included in the enriched gene set include one-component (49) and two-component system (50) genes, which show a similarly disparate regulatory profile as the alternative sigma factors (Figure 1D–E; Tables S1 & S2).

#### DC induces an acute stress response

Among the most highly activated genes in the dataset are those with known roles in acute stress responses, including genes that mitigate protein misfolding (enriched GO terms: unfolded protein binding; protein folding) (Table S2; Figure 1B–C). Genes in this class include the chaperones *groEL*, *groES*, *htpG*, *dnaK*, *clpB*, and a small Hsp20-family protein (*ptos_002388*). *msrB*, which repairs oxidatively-damaged methionine residues, is strongly induced suggesting that DC treatment causes oxidative stress. Indeed, transcription of the *katB* catalase (*ptos_001055*), superoxide dismutase (*ptos_002119*), peroxide stress protein YaaA (*ptos_003649*) and glutathione peroxidase (*ptos_003459*) are activated, as are DNA starvation/stationary phase protection protein (*dps; ptos_001118*), and universal stress protein (*usp*; *ptos_002049*) (enriched GO terms: response to oxidative stress; cellular oxidant detoxification; and cellular homeostasis) (Table S2, Figure 1B–C). We further observed induction of all three genes involved in production of inorganic polyphosphate (*ptos_002410, ptos_002960, ptos_003236*), which is known to confer general protective effects during stress exposure in a variety of bacterial taxa (51–54).

#### Evidence for a metabolic shift upon DC exposure

By far, the most significantly enriched functional categories in the down-regulated set of genes involve translation (enriched GO terms: ribosome, translational elongation, aminoacyl-tRNA ligase activity, tRNA aminoacylation for protein translation). Expression of ribosomal proteins, ribosomal accessory factors, and aminoacyl tRNA synthetases is uniformly lower (by 2–50 fold), with few exceptions (Figure 1B–C, Tables S1 & S2). This effect resembles gene expression changes induced by the stringent response (55), which slows growth and facilitates cellular adaptation to nutrient or energy limitation. The wholesale downregulation of protein expression machinery is accompanied by reduced expression of genes required for cell growth and division (enriched GO terms: regulation of cell shape, cell wall organization, peptidoglycan biosynthetic process, lipopolysaccharide biosynthetic process, cell envelope) (Tables S1 & S2). Other significantly down-regulated biosynthetic pathways (FDR q-value < 0.05) include genes required for production of cobalamin, ribonucleosides, pseudouridine, pantothenic acid, queuosine, arginine, and coenzyme A.

While the transcriptomic data provide evidence that deoxycholate treatment results in a large-scale downregulation of anabolic metabolism, expression of select metabolic enzymes is activated. Among the most notable of these responses is the transcriptional activation of genes involved in glutamate metabolism (enriched GO term: glutamate metabolic process) (Table S2). Specifically, DC treatment induces expression of asparagine synthase B (*asnB; ptos_002403*), glutaminase (*glsA; ptos_00368*), genes in the histidine utilization pathway (*ptos_003734–3738*), and glutamate dehydrogenase (*gdhA; ptos_003163*), all of which catalyze production of glutamate from various substrates. The expression of glutamate decarboxylase (GAD, *ptos_000367*), which converts glutamate to γ-amino butyric acid (GABA), is also activated. This reaction consumes a proton and is thought to contribute to neutralization of an acidified cytoplasm (56). Glutamate decarboxylase is associated with tolerance of acid and bile acid stress in many bacteria, including *Bacteroides* species (57–59) .

A second set of upregulated metabolic genes have predicted roles in citrate/carboxylate metabolism (enriched GO term: tricarboxylic acid cycle) including the gene cluster *ptos_003272–3274* (citrate synthase, isocitrate dehydrogenase, and aconitase) and a succinyl-CoA ligase system (*ptos_001896–1897*). Activation of lactate/malate dehydrogenase (*ptos_000443*) and L-lactate permease (*ptos_001319*) expression by approximately 50-fold in the presence of DC indicates a shift in carboxylic acid metabolism in *B. fragilis* during bile stress. Finally, transcription of multiple enzymes comprising the pentose phosphate pathway (enriched GO term: pentose-phosphate shunt) including *gnd*, *zwf*, and *pgl* (*ptos_1536–1538*), *fba* (*ptos_002818*) and *rpiA* (*ptos_003205*) are activated by DC. Other genes in this pathway including *rpiB* (*ptos_001356*) and *ptos_002671* and *ptos_002867* are repressed by DC (Figure 4). This pathway produces key substrates for anabolic metabolism and also yields reducing equivalents.

#### Membrane transport systems

Transcription of the F_O_F_1_ ATP synthase operon (*ptos_001821–1829*) is activated upon DC exposure (enriched GO terms: proton motive force-driven ATP synthesis), suggesting that DC compromises energy production in *B. fragilis*, perhaps by affecting membrane permeability. Indeed, expression of multiple genes with predicted roles in gated ion transport (enriched GO terms: monoatomic ion gated channel activity) (Table S2) is activated by DC including ion channels *ptos_003102* and *ptos_000442*, mechanosensitive ion channels *ptos_002809*, *ptos_004068*, *ptos_000516*, *ptos_000659*, *ptos_000815*, and the H(+)/Cl(−) exchange transporter *ptos_001050*. Overall, we observe a variable pattern of activation and repression of annotated ion transporters with the highest level of activation ( ≈20-fold) for a Na+/H+-translocating pyrophosphatase family protein, *ptos_000210* (60, 61), and transporters with predicted roles in sulfate (*ptos_002300*) and zinc transport (*zup; ptos_002150*). The large effect of DC treatment on ion transport gene transcription provides evidence of general dysregulation of cellular ion homeostasis.

**Figure 2:**
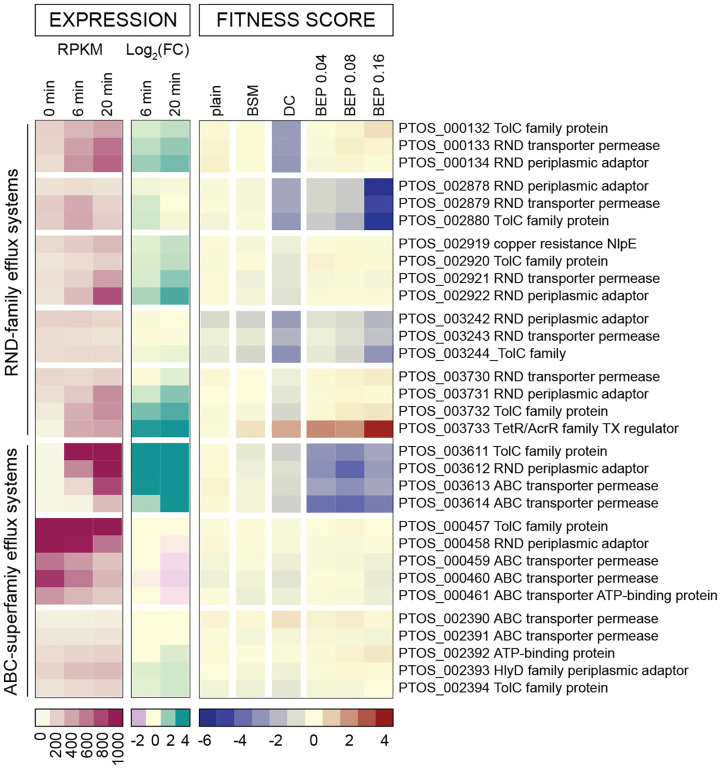
Multiple efflux systems differentially contribute to *B. fragilis* fitness in the presence of purified bile acid and crude bile conditions. Transcript abundance and fitness scores of TolC-family efflux system operons that are either transcriptionally regulated in the presence of DC or contribute to fitness in at least one bile condition. A similar heat map of all 23 TolC-containing operons is presented in Figure S2. Transcript abundance is presented as reads per kilobase per million reads (RPKM) and regulation is indicated by the log_2_(fold change) of gene counts per million compared to untreated cells (0 min). Color scales representing absolute expression, fold change in expression, and mutant fitness scores are presented below each type of data.

In addition to regulation of genes involved in ion transport processes, DC induces changes in expression of RND- and ABC-family tripartite efflux systems, which can transport harmful xenobiotic compounds such as bile acids out of the cell (62). Tripartite efflux systems are comprised of an inner membrane permease, a TolC-family protein in the outer membrane, and a periplasmic adapter protein that bridges the inner and outer membrane proteins (63). In RND-family systems, the inner membrane permease harnesses ion motive force to power efflux through a TolC-family outer membrane protein; ABC-family export systems leverage the chemical energy from ATP-hydrolysis to secrete molecules from the cell via a TolC-family protein (62). Tripartite efflux systems have been associated with bile efflux and resistance in many resident and pathogenic gut microbes (26). A total of 23 TolC-family exporter systems are encoded by *B. fragilis P207* and, like other systems described above, expression of this gene family is variable with some TolC exporters strongly activated by DC and others repressed (Figure 2 and Figure S2). The operon encoding a TolC-family transport system, *ptos_003611–003614*, contains the most highly activated genes in the *B. fragilis* P207 genome upon DC exposure (>200-fold induction by 20 minutes) (Figure 1; Table S1). Pumbwe and colleagues previously measured transcriptional changes in 13 RND-family efflux systems in *B. fragilis* 638R the presence of bile salts (64). While congruence of these data with our transcriptional dataset is low, both studies reveal similar regulation of the transport operon *ptos_003730–33* (*bme5* in (64)) in response to bile salt treatment.

We conclude that the global transcriptional response to DC exposure is consistent with large-scale remodeling of the physiological status of the cell to slow growth, shift carbon and energy metabolism, and to mitigate a constellation of cellular stresses that arise due to bile exposure. Although these transcriptomic data are informative, the presence of over 1000 regulated transcripts in our dataset posed challenges in assigning the contribution of specific genes or pathways to *B. fragilis* fitness in bile. As such, we sought to extend our transcriptomic study by implementing a genetic approach to directly identify genes that contribute to *B. fragilis* P207 fitness in the presence of DC and a crude bile extract. To this end, we developed a randomly barcoded pool of *B. fragilis* P207 Tn-*Himar* mutants and utilized random barcoded transposon sequencing (RB-TNSeq or BarSeq) (65, 66) to identify insertional mutants with enhanced or diminished fitness in the presence of DC.

### Transposon mutagenesis of B. fragilis P207 defines a set of candidate essential genes

To enable genetic analysis of *B. fragilis* P207 physiology, including bile resistance, we generated a pool of >50,000 barcoded Tn-*Himar* insertional mutants. Mapping the insertion sites in the pool identified 49,543 reliably mapped barcodes at 43,295 distinct sites in the genome. This *B. fragilis* Tn-*Himar* pool contains a median of 8 mutant strains per gene, and at least one insertion in the central region of 87.9% of protein coding genes (Table S3). In parallel, we produced a collection of 889 Tn-*Himar* mutant strains that were arrayed and individually mapped (Table S4). This arrayed collection contains strains carrying transposon insertions in approximately 14% of the predicted genes in the *B. fragilis* P207 genome.

We employed a probabilistic approach that relies on hidden Markov models (HMMs) to quantify gene essentiality in mutant libraries (67), and identified 310 candidate essential genes and 258 genes for which Tn-*Himar* insertions are predicted to yield growth defects in BHIS medium (Table S5). We further used a Bayesian (Gumbel) approach (68) to assess genes essentiality (Table S5). As less than one-quarter of TA dinucleotide sites carry insertions in the pool, these candidate essential lists are likely incomplete. Nonetheless, expected essential genes including those with key roles in cell division, cell envelope biogenesis, DNA replication, transcription, and translation were common to both the Gumbel and HMM essential lists.

We note several candidate essential genes that we found to be of particular interest. Transposon insertions were not recovered in one of the two RelA/SpoT paralogs (*ptos_000629*), which regulate levels of the alarmone ppGpp and metabolic adaptation to stress (55). Similarly, in the closely related species *Bacteroides thetaiotaomicron* one of the RelA/SpoT paralogs is also essential (69). Other notable candidate essential genes include a predicted two-gene operon encoding a type III restriction enzyme (*ptos_000974*) and a type III adenine DNA methyltransferase (*ptos_000975*), and the *rokA* carbohydrate kinase (*ptos_000462*). *rokA* has been successfully deleted in a derivative of *B. fragilis* strain 638R (70), but is classified as a candidate essential gene in P207 in our growth conditions based on HMM analysis of insertion data. A *upxY* family transcription antiterminator (*ptos_002531*) is classified as essential by HMM. The orthologous gene in *B. fragilis* 638R (BF638R_2798) was also classified as essential in a global Tn-seq study (71). Unusually, this *upxY* paralog is not adjacent to a corresponding *upxZ* anti-antiterminator, as is typical in *Bacteroides* capsular polysaccharide (CPS) loci (72). Rather, *ptos_002531* is the first gene in a putative 28 gene operon that encodes the machinery to synthesize the large capsule EPS elaborated on a fraction of cells in a population (73). Within this large predicted operon, insertion in all downstream genes are recovered, though a gene of unknown function (*ptos_002544*) and in a glycosyltransferase family 4 gene (*ptos_002546*) are scored as growth defective based on the distribution of insertions recovered (Table S5).

### Barcoded transposon mutagenesis identifies genes that influence *B. fragilis* fitness in bile

To identify *B. fragilis* P207 genes that specifically affect fitness in bile by BarSeq, it was first necessary to define treatment concentrations that resulted in intermediate growth inhibition of the parental P207 strain (Figure S1). By assessing fitness of Tn-*Himar* mutants in bile concentrations in which wild-type P207 was inhibited at an intermediate level, we expected to simultaneously identify strains that are more sensitive and more resistant to treatment. Growth in the presence of 0.01% bile salt mixture (BSM) or DC resulted in a 20–30% reduction in the terminal density of *B. fragilis* P207, respectively. Growth in crude porcine bile extract was variable, likely due to day-to-day variation in the preparation and solubilization of the crude extract (Figure S1B). The barcoded Tn-*Himar* mutant pool was therefore cultivated in BHIS broth with either 0.01% DC, 0.01% BSM, or a range of BEP concentrations (0.04, 0.08 and 0.16%). In parallel, we cultivated the pool in plain BHIS medium to differentiate strains with general growth defects from those with bile specific growth defects. Once cultures approached terminal density, they were diluted back into fresh media. This serial passaging approach amplified fitness differences between mutant strains; barcode abundances were evaluated after the first and second passages. Fitness scores for each gene in each condition were calculated using the approach of Wetmore et al. (66) and are presented in Table S6. Briefly, fitness scores represent the composite fitness advantage or disadvantage (log_2_ change in relative abundance) of all strains bearing insertions in a particular gene relative to a control condition. Thus, negative fitness scores indicate genes that support fitness in the presence of bile, whereas positive fitness score indicate genes that that have a negative effect on fitness in bile.

Principal component analysis of the genome-scale fitness data showed high experimental reproducibility between each biological replicate (Figure S3). The fitness profiles of the P207 mutant pool cultivated in DC and BSM were more like each other than porcine bile extract, which was expected given that BSM is a 1:1 mixture of cholate and DC. Exposure to DC had a greater fitness impact on *B. fragilis* than exposure to an equivalent concentration of BSM. Finally, we observed more variability in the fitness profile of *B. fragilis* exposed to BEP, consistent with growth variability we observed in this condition. To validate our Barseq fitness measurements, we selected mutant strains that harbored transposon insertions in genes identified as fitness factors in the BarSeq dataset from the arrayed collection of individually mapped mutants (Table S4). Overall, the growth of these individual mutant strains was consistent with the fitness scores for the corresponding genes derived from BarSeq (Figure S4), lending confidence to the genome scale data set.

**Figure 3:**
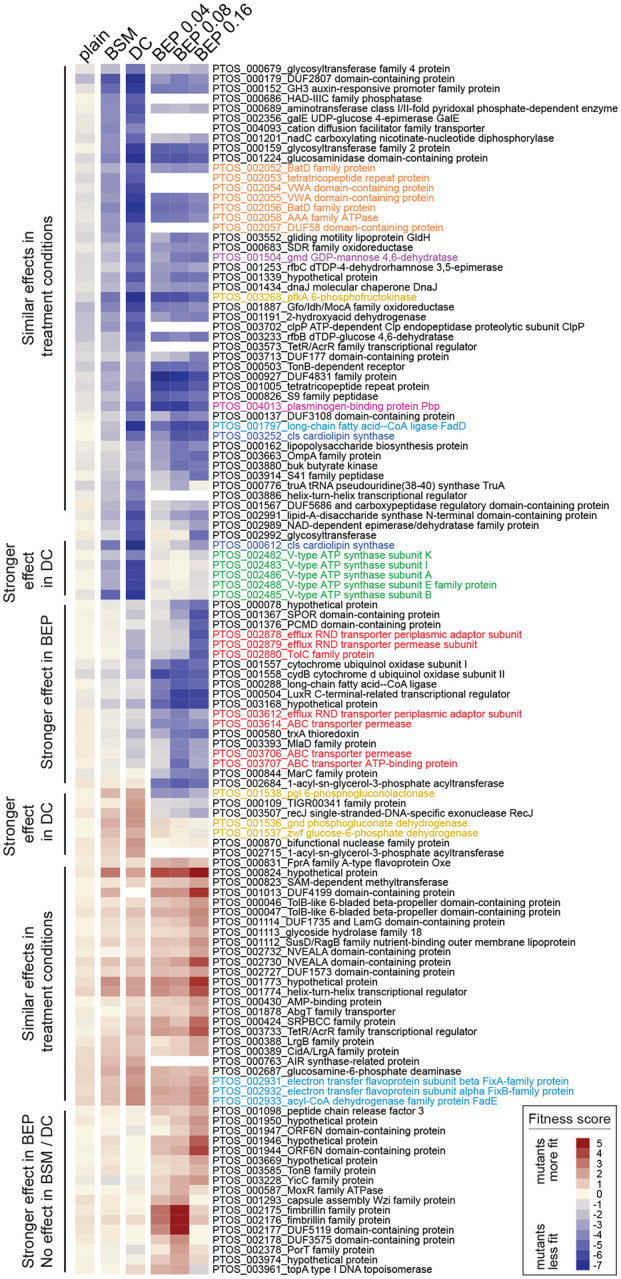
Genes that contribute to *B. fragilis* fitness in the presence of purified bile acids and crude bile have diverse metabolic, stress response, and cell envelope functions. Heat map of fitness scores for the 122 genes that are significant determinants of *B. fragilis* fitness in at least one treatment condition (see Materials and Methods for significance thresholds); treatment conditions are arranged in columns and genes in rows. Composite fitness scores for genetic mutants grown in plain BHIS medium without bile are labeled (plain). Mutant strain growth was measured in BHIS containing 0.01% (w/v) bile salt mixture (BSM), 0.01% (w/v) deoxycholate (DC), and 0.04%, 0.08%, and 0.16% bile extract of porcine (BEP). Gene-level fitness scores were hierarchically clustered; gene arrangement was manually adjusted to group genes presumed to be in operons. White blocks indicate genes with insufficient barcode counts in the reference condition of a particular experiment to calculate a fitness score. Colored gene names highlight select functional categories discussed in the text: red – efflux systems, green – V-ATPase operon, dark blue – cardiolipin synthase genes, light blue – lipid metabolism, mustard – central carbon metabolism, orange – Bat aerotolerance operon, fuchsia – plasminogen-binding protein (Pbp), and purple – *gmd* protein glysosylase.

Genes that impacted fitness in each bile condition were defined as those with average fitness scores less than −4 or greater than +1.5 after the second passage, excluding genes that affected growth in plain BHIS medium. Using these criteria, 14 genes were identified that are determinants of *B. fragilis* P207 fitness in 0.01% BSM, 63 in 0.01% DC and 89 in at least one concentration of BEP. Together this represents a set of 122 genes that significantly influence growth in at least one bile treatment condition. Clustering this gene set based on fitness scores revealed high overlap in groups of genes that positively or negatively contributed to *B. fragilis* fitness across all conditions, and further revealed a set of genes that contributed to fitness in a specific bile condition (Figure 3). No gene was identified as a BSM-specific fitness factor. Thus, in further discussion we primarily consider BarSeq data from DC and crude bile extract.

### Multiple gene classes contribute to *B. fragilis* fitness in the presence of bile

Genes that function in cell envelope biosynthesis, energy metabolism, membrane transport, and stress responses are among the major bile fitness factors. We present results from each of these functional classes.

#### Cell envelope biosynthesis

Bile salts are detergent-like molecules that can disrupt membranes, so it was expected that genes involved in biosynthesis of components of the *B. fragilis* cell envelope would impact fitness in the presence of bile. The phospholipid, cardiolipin, has been implicated in adaptation and/or resistance of bacteria to envelope stress conditions including high osmolarity (74) and bile stress (75, 76). *B. fragilis* encodes two predicted cardiolipin synthases, *ptos_000612* and *ptos_003252*, and disruption of either of these genes resulted in sensitivity to bile. The functions of these genes are therefore not entirely redundant under the tested conditions. Notably, disruption of *ptos_00612* resulted in a severe fitness defect in DC but only a modest defect in crude bile extract, while strains harboring disruptions in *ptos_003252* had similar fitness defects in both DC and crude bile (Figure 2, Table S6).

Many proteins and lipids of the *B. fragilis* cell envelope are heavily glycosylated, and mutation of select genes involved in envelope glycosylation is known to impact colonization of mammalian hosts (77). *gmd* (*ptos _001504*; GDP-mannose 4,6-dehydratase) and *fcl* (*ptos_001503*; GDP-L-fucose synthase) function in a protein O-glycosylation that is important for host colonization (78, 79). Transposon insertions in *gmd* resulted in a severe fitness defect when *B. fragilis* P207 was cultivated in the presence of DC and crude bile extract; the consequence of disrupting GDP-L-fucose synthase (*ptos_001503, fcl)* was less severe (Figure 3, Table S6). A target of the *gmd-fcl* glycosylation pathway is a glycosyltransferase encoded by *ptos_002992* (80); strains with insertions in this gene also had reduced fitness in bile. Tn-*Himar* insertions in several other genes with predicted functions in biosynthesis of capsular polysaccharide (CPS), lipopolysaccharide (LPS) or other surface polysaccharides (e.g. *ptos_000679, ptos_000683, ptos_000686, ptos_000689, ptos_000159, ptos_000162, ptos_001253, ptos_001293, ptos_002991, ptos_002992, ptos_003233*) resulted in sensitivity to bile and bile acid treatments. Among characterized *B. fragilis* surface-exposed lipoproteins (81, 82), the highlyexpressed plasminogen-binding protein (Pbp; *ptos_004013*; (81)) conferred resistance across all conditions and was among the most important DC resistance factors in our dataset (Figure 3, Table S6). Disruption of the *pbp* ortholog in *B. thetaiotaomicron*, *BT2844*, also resulted in lower fitness when strains were grown in the presence of a range of bile compounds (65).

#### Carbon and energy metabolism

Disruption of long chain fatty acid-CoA ligase (*fabD; ptos_001797*), which activates long-chain fatty acids for either synthesis or beta oxidation, resulted in sensitivity to all treatment conditions. *fadE* and *fixAB* (*ptos_002931–33*) execute the first step in beta oxidation, and disruption of these genes conferred a fitness advantage in the tested conditions (Figure 3), supporting a model in which activation of fatty acids by *fabD* for anabolic, rather than catabolic processes, enhances bile acid resistance. Two additional long chain fatty acid-CoA ligases are annotated in the P207 genome: *ptos_000288* and *ptos_003405*. Insertions in *ptos_000288* resulted in fitness defects in crude bile extract, but not in DC, while insertions in *ptos_003405* had little impact under either condition (Table S6).

**Figure 4:**
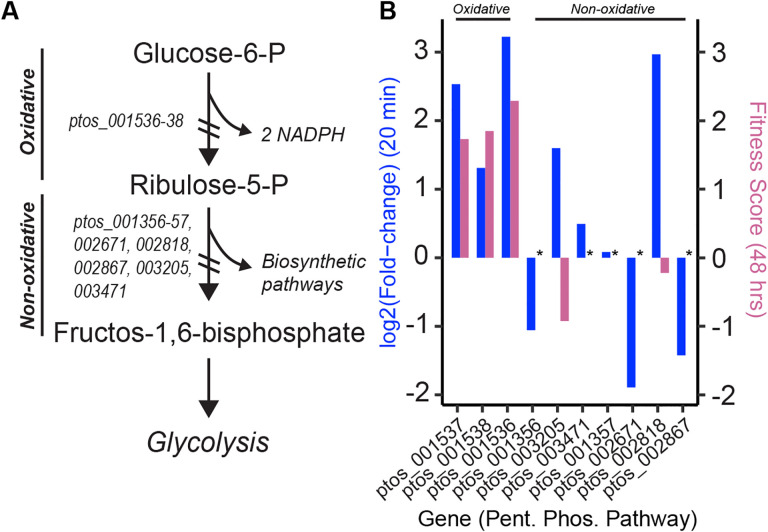
Transcriptional regulation and fitness contributions of genes in the pentose phosphate pathway in the presence of deoxycholate. (**A)** Schematic overview of the oxidative and non-oxidative phases of the pentose phosphate pathway. Locus numbers of the genes encoding the enzymes involved in each phase are indicated. (**B)** For each gene in the pathway, change in mean transcript level after 20 minutes of exposure to DC (blue; left y-axis) and mean fitness score after second passage in DC (red; right y-axis) are presented. Expression of genes in the oxidative phase is enhanced in the presence of DC, yet strains in which these genes are disrupted have a fitness advantage.

Disruption of phosphofructokinase (*pfkA*; *ptos_003268*) resulted in severe fitness defects in all bile treatment conditions. PfkA catalyzes the committed step in glycolysis, which converts glucose to pyruvate and generates ATP via substrate level phosphorylation. Glucose can also be shunted to the pentose phosphate pathway, which generates NADPH and pentose sugars, and strains harboring insertions in genes comprising the oxidative branch of the pentose phosphate pathway (*zwf*, *pgl* or *gnd*; *ptos_001536–38*) have a fitness advantage in the presence of DC (Figures 3 & 4, Table S6). From these results, we infer that an active glycolytic pathway is more advantageous than shunting glucose to the pentose phosphate pathway in the presence of DC though, surprisingly, *zwf*, *pgl* and *gnd* are all transcriptionally activated upon acute DC exposure (Figure 4, Table S1). Both the beta-oxidation and pentose phosphate pathways generate reducing equivalents, and disruption of genes in either of these pathways is advantageous in the presence of bile.

**Figure 5:**
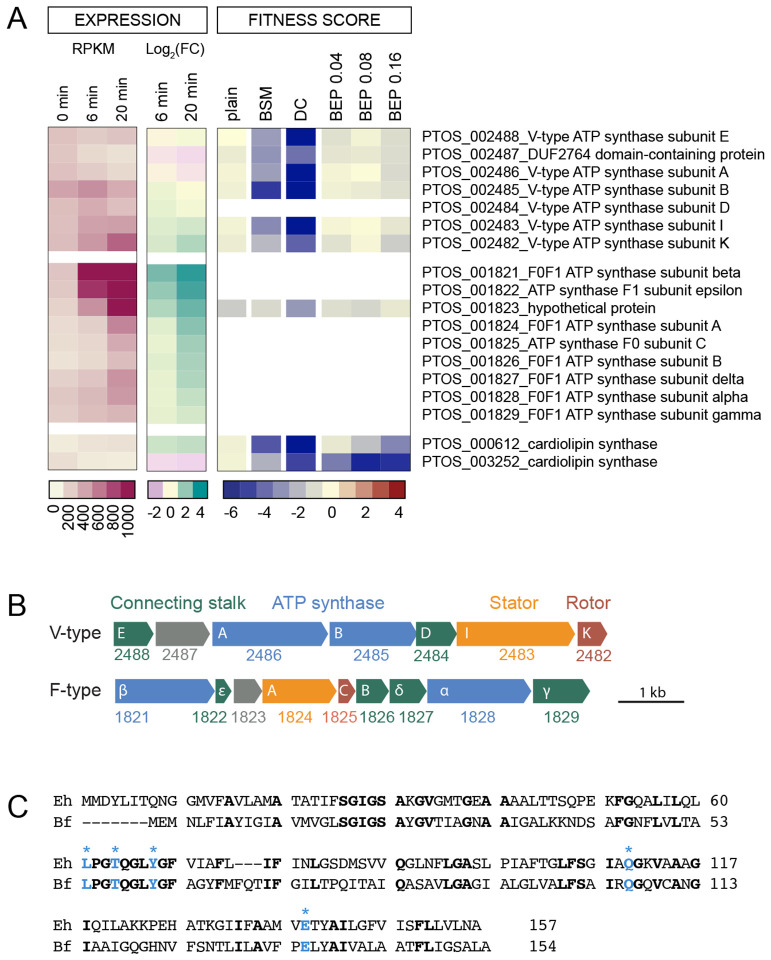
B*. fragilis* encodes two rotary ATP synthase / ATPase systems; the V-type system bears conserved residues for sodium ion translocation and is a critical fitness factor in deoxycholate. **(A)** Expression level and fitness scores for genes in the V-type and F-type ATP synthase / ATPase operons, presented as in Figure 2. White blocks indicate genes with insufficient barcode counts in the reference condition to calculate a fitness score. Color scales representing absolute expression (RPKM), fold change in expression, and mutant fitness scores are presented below each type of data. **(B)** Operon structure of F-type and V-type ATPase systems. PTOS gene locus numbers are below gene outlines; annotated subunit names are in white. The cytoplasmic subunits responsible for ATP synthesis/hydrolysis are blue, the transmembrane rotary subunits that translocate ions are red, the transmembrane stator subunits are orange, the stalk subunits that connect the enzymatic complex to the membrane complex are green, and unannotated genes are grey. The V-type system has fewer connecting accessory subunits than the F-type system and other characterized bacterial V-type systems (107). **(C)** Protein sequence alignment of the K subunits of *B. fragilis* (Bf) and the sodium ion translocating *Enterococcus hirae* (Eh) V-ATPases. Identical residues are bold. Residues experimentally determined to coordinate sodium ions in *E. hirae* (85) are highlighted in blue with asterisk.

Strains with insertions in the V-type ATPase gene cluster (*ptos_002482-ptos_002488*) were among a limited group that had highly reduced fitness in DC and no defect in the tested concentrations of porcine bile extract. In fact, the fitness defects of these V-type ATPase mutants were among the most extreme in the entire DC dataset (Figure 3, Table S6). The primary structures of V-type ATPase proteins PTOS_002482-PTOS_002488 have features that distinguish them from the *B. fragilis* P207 F_O_F_1_-type ATPase proteins (PTOS_001821–001829), and that are consistent with complexes that either couple sodium-motive force to the generation of ATP, or hydrolyze ATP to pump Na^+^ from the cell. Specifically, the inner membrane subunit that constitutes the ion translocating rotor has all of the residues that have been structurally identified to coordinate and translocate Na^+^ ions (Figure 5 (83–85))

#### Stress response systems

Tn-*Himar* insertions in several genes implicated in stress responses resulted in sensitivity to all treatments, including the *bat* genes (*ptos_02052–58*), which have a reported role in aerotolerance (86). Fitness defects were also observed in all bile conditions in strains with disrupted *dnaJ* (*ptos_001434*), which encodes a protein chaperone important for maintaining proteostasis during stress. Similarly, disrupting the serine protease gene, *clpP* (*ptos_003702*), which fosters protein quality control during stress, resulted in severe fitness defects in the presence of DC; the phenotype of strains with insertions in the ATP binding subunit of the Clp protease, *clpX* (ptos_003701), were less severe (Figure 3, Table S6).

#### Efflux systems

Efflux is a well-established mechanism of bile-tolerance (26). TolC-family outer membrane proteins can work in conjunction with RND and ABC family transport systems to enable efflux of a broad range of substrates (62, 87) including bile acids. Previous work in *B. thetaiotaomicon* identified a TolC-type bile-induced efflux system, BT2792-BT2795, that specifically contributed to fitness in the presence of multiple bile acids (65). *B. fragilis* P207 does not encode an orthologous system, but we identified multiple tripartite TolC-containing efflux systems that contribute to fitness in DC and BEP, likely in a redundant fashion (Figure 2). The overall repertoire of tripartite efflux genes that support growth/survival in DC and BEP is similar, though reduced fitness due to disruption of *ptos_000132–34* is specific to DC.

The TolC-family protein encoded by *ptos_003611* provides an example of a gene that is transcriptionally regulated by DC but that has little impact on fitness when disrupted. In fact, *ptos_003611* is the most highly induced gene in the DC transcriptomic data set (Figure 1, Table S1) yet the fitness consequence of disrupting this gene and adjacent transport genes in its operon is minor in DC, where disruption of other efflux systems had a larger impact on fitness (Figure 2). Similarly, transcription of the transport genes *ptos_003730–32* is activated by DC treatment but insertions in this locus do not impact fitness in DC. However, Tn-*Himar* insertions in *ptos_003733*, which encodes a transcriptional repressor adjacent to *ptos_003730–32* confers a fitness advantage in the presence of DC and BEP (Figure 2). From this result, we infer that de-repression of the *ptos_003730–32* efflux system via disruption of its transcriptional repressor supports growth and/or survival in DC and BEP. Overall, there is little correspondence between transcriptional regulation of efflux genes and their contribution to fitness in our BarSeq assay.

#### Specific fitness factors in crude porcine bile

Insertions in two efflux systems (*ptos_002878–80* and *ptos_003611–14*) and in components of the predicted lipopolysaccharide export system (*ptos_003706–07*) are more detrimental to *B. fragilis* fitness in BEP than in DC (Figures 2 & 3). Additionally, strains with disruptions in a cluster of genes of unknown function (*ptos_001944, ptos_001946, ptos_001947* and *ptos_001950*) and two genes encoding fimbrillin family proteins (*ptos_002175–76*) resulted in a specific fitness advantage in crude bile.

## DISCUSSION

### A new resource for the study of *B. fragilis* physiology

We have developed *B. fragilis* isolate P207 as a new model system to investigate genes and pathways that contribute to *B. fragilis* fitness in conditions encountered in the inflamed mammalian gut. Other well-studied strains of *B. fragilis* have been isolated from infection sites where this species was an opportunistic pathogen (e.g. NCTC9343 from an appendix abscess, and 638R from an abdominal abscess); strain P207 was isolated from the distal bowel of an ulcerative colitis patient who experienced inflammation of the ileal-rectal pouch, or pouchitis (24). A diverse pool of barcoded *B. fragilis* P207 Tn-*Himar* mutants was produced, which can be used to interrogate strain-level fitness at the genome scale in any condition of interest. To our knowledge, this is the first barcoded transposon mutant library in *B. fragilis*, a species that is typically less amenable to molecular genetic analysis than other well-studied *Bacteroides* species. We have further generated an arrayed collection of individually mapped transposon insertion mutants that captures ≈14% of genes in the genome and that can be used for directed studies of individual *B. fragilis* genes.

Given the major role of bile acids in shaping the gut microbiota and reported shifts in the bile acid profile of the inflamed gut, we used this new genetic resource to identify genetic and physiological factors that contribute to *B. fragilis* P207 bile acid tolerance. We observed large-scale changes in gene expression wherein over one-quarter of transcripts changed significantly in abundance within 20 minutes of exposure to DC. These data evidence a physiologic shift involving a reduction of protein synthesis capacity, and enhanced stress mitigation processes (Table S1). Enhanced transcription of stress response genes is consistent with other enteric microbes for which bile responses have been studied (26), and functional genetic analyses of DC and BEP tolerance/resistance using our barcoded library provided additional support for the importance of stress mitigation factors in the presence of bile: chaperones (*dnaJ*), proteostasis factors (*clpP*) and the BAT system all contribute to fitness (Table S6). Additional genetic factors that contributed to fitness are discussed below.

A limited number of mutants exhibited enhanced fitness in our treatment conditions including genes involved in the pentose phosphate pathway and genes that function in the committed steps of fatty acid beta-oxidation. Thus, the oxidative branch of the pentose phosphate pathway and lipid oxidation are apparently detrimental in the tested conditions. We have further identified many genes of unknown function that are important for survival in the presence of bile; future studies of these genes may reveal new molecular mechanisms of bile acid resistance.

### Cardiolipin and bile acid resistance

The anionic phospholipid, cardiolipin, is not typically an essential component of bacterial membranes but plays a critical role in supporting function of trans-envelope protein complexes including respiratory complexes and ATP synthase (88–92). *B. fragilis* encodes two cardiolipin synthase genes, *ptos_000612* and *ptos_003252*, which have distinct contributions to fitness in the presence of bile. Several lines of evidence indicate that cardiolipin stabilizes bacterial membranes in the presence of bile acids. For example, fitness defects have been reported for cardiolipin synthase mutants in *Entercoccus faecium* exposed to bile (76), and elevated levels of cardiolipin have been reported in *Lactobacillus* adapted to growth in sub-lethal concentrations of bile salts (75). In addition, phospholipid vesicles containing high fractions of cardiolipin are more resistant to solubilization by bile salts than vesicles with low cardiolipin (75).

Generally, cardiolipin facilitates the dynamic movement of proteins in the lipid bilayer, stabilizes multi-protein complexes, and acts as a proton trap to facilitate transfer of protons exported during electron transport to the ATP synthase (90, 93–95). This role as a proton trap is important in the case of bile acid exposure because membrane stresses are associated with increased proton leakage, and dissipation of proton motive forces (PMF). Indeed, exposure to membrane decoupling agents leads to elevated levels of cardiolipin (96), providing evidence that this this lipid may be regulated to mitigate loss of membrane potential during stress. DC degenerates PMF at concentrations found in the distal bowel of healthy patients, which are similar to the DC concentrations used in this study (27, 35). The collapse of proton gradients across the bacterial cell membrane compromises the function of ATP synthase(s), which can both harness the potential energy of trans-membrane ion gradients to synthesize ATP or use the chemical energy stored in ATP to pump ions across the membrane against a chemical gradient (depending on the concentrations of ATP and ions).

### The contribution of membrane bioenergetic systems to fitness in bile

*Bacteroides* species encode two distinct ATP synthase systems: a standard F_0_F_1_ system (PTOS_001821–29) that utilizes a proton motive force to generate ATP, and a second V-type ATPase (PTOS_002482–88) that has the conserved residues to coordinate and translocate sodium ions (Figure 5) (83). We recovered very few strains with transposon insertions in the F_0_F_1_ ATP synthase genes (Table S5) and were therefore unable to evaluate the fitness of F_0_F_1_ mutants. However, the genes encoding the V-type system were dispensable under standard cultivation conditions. Thus strains with transposon insertions in these genes were sufficiently represented in the pool to assess their contribution to fitness by BarSeq. The V-type system was essential in the presence of DC, as strains with insertions in these genes became nearly undetectable after serial cultivation. In eukaryotic cells this class of ATPases has been characterized as proton pumps that function to acidify vacuoles, but V-type systems in bacteria (also called A-type) can couple ion motive forces to ATP synthesis (e.g. *Thermus thermophilus* (97)), or expend ATP to pump ions from the cell to maintain cellular homeostasis (e.g. in *Enterococcus hirae* (98)). The function of the V-type system in *Bacteroides* is not known. Considering the detrimental effect of bile acids on membrane integrity, the presence of conserved Na^+^-coordinating residues in the system, and the fact that membranes are less permeable to Na^+^ than H^+^, we hypothesize that the V-type system functions primarily as an ATP synthase that harnesses sodium motive force to support ATP production when membrane disruptors such as DC are present. ATP synthases that can translocate Na^+^ rather than H^+^ are potentially advantageous in conditions where proton gradients can become compromised.

The operon encoding the V-type ATP synthase is broadly conserved in Bacteroidetes including in *Porphyromonas gingivalis* where it is reported to be upregulated in the presence of sapeinic acid, a host-derived lipid that disrupts bacterial membranes (99). Expressing multiple ATPase/synthase systems likely enables bacteria to leverage distinct ion gradients to support fitness in niches where proton gradients may be unreliable due to extreme pH, high temperature or an abundance of membrane disrupting chemicals (83). Notably, *B. fragilis* encodes a sodium pumping oxidoreductase, Nqr, that facilitates the establishment and maintenance of Na+ gradients during electron transport; Nqr accounts for about 65% of the NADH:quinone oxidoreductase activity of the cell (100) indicating that the maintenance of sodium gradients is an important aspect of *B. fragilis* physiology. Notably, sodium concentrations can become elevated in the inflamed the gut of UC patients (101), and bacteria that can leverage elevated sodium levels for energy production may have an growth advantage.

To our knowledge the *Bacteroides* V-type ATPase/synthase has not been functionally characterized. It has features of other V-type systems - such as a duplicated c/K subunit that forms the ion-conducting pore - but has a distinct number of subunits compared to characterized ATPase/synthase operons. In addition to the cytoplasmic alpha and beta subunits that form the catalytic domain, the ion translocating k subunit, and the stator subunit I, the *Bacteroides* V-type operon encodes only three additional proteins to coordinate ion translocation with ATP synthesis/hydrolysis, which is fewer than other bacterial V-type ATPase systems (102). The second gene in the *Bacteroides* V-ATPase operon, *ptos_002487*, is annotated as DUF2764. This conserved protein is exclusively bacterial and most commonly found in the order Bacteroidales (45). The genes comprising this unique V-ATPase genetic element are clearly important fitness determinants in the secondary bile acid, DC, and merit further study.

### Correspondence between genes that are transcriptionally regulated and genes that contribute to fitness

**Figure 6:**
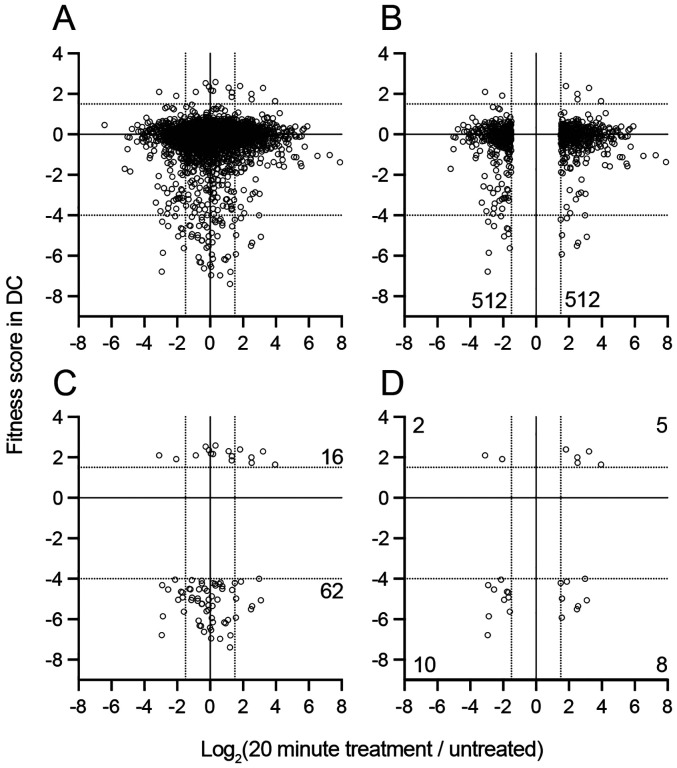
Genes that are transcriptionally regulated by DC treatment are weakly correlated with genes that determine fitness in medium containing DC. Log_2_(fold change) in transcript levels before and after 20 minutes of exposure to 0.01% DC (x-axis) versus gene-level fitness scores after cultivation in 0.01% DC (y-axis). Each point is a gene, for **(A)** all measured genes, **(B)** genes with significant differences in transcript, **(C)** genes with significant fitness scores, or **(D)** significant in both data sets. Dotted lines indicate significance thresholds on each axis. Numbers on graph indicate the number of genes in each region of the graph that meet the selection criteria.

We have used two complementary approaches to identify genes and pathways that impact physiology and fitness of *B. fragilis* strain P207 in purified bile acids and crude bile. Transcriptomic analysis during acute DC exposure provided a global view of how a secondary bile acid impacts gene expression in *B. fragilis*. Though one may expect some degree of overlap between gene sets that were regulated by DC and genes that contributed to fitness in bile-containing media, the transcriptomic and barseq data sets were surprisingly incongruent (Figure 6). Genes that impacted fitness were generally not transcriptionally regulated. Likewise, genes regulated by DC did not typically impact fitness when disrupted. Even among genes that were transcriptionally regulated and that contributed to fitness in bile, the connection between the directionality of regulation and fitness was often difficult to rationalize. For example, select genes were transcriptionally activated by DC exposure, but conferred a fitness advantage when disrupted including genes in the pentose phosphate pathway (Figure 4, Table S1 & S6). This lack of correlation between transcriptional response and functional genetic analyses has been noted in omics studies of bile resistance in unrelated species (76). The RNA-seq and Tn-seq approaches that we have applied to study bile acid responses and bile resistance give complementary insight into the physiological response to and genetic requirements for *B. fragilis* to contend with bile acids in the gut environment and open multiple avenues for future investigation of *B. fragilis* and other microbial blooms in the inflamed gut.

## MATERIALS AND METHODS

### Bacterial strains and primers.

Strains and primers used in this study are listed in Table S7. All primers were synthesized by Integrated DNA Technologies (Coralville, IA, USA).

### Growth media.

*B. fragilis* strain P207 was grown in BHIS medium (37 g/L Bacto^™^ Brain Heart Infusion (Becton, Dickinson and Company), 10 g/L Yeast Extract (Fisher BioReagents), 0.5 g/L L-cysteine (Sigma), supplemented after autoclaving with 5 ug/ml hemin and 1 ug/ml vitamin K. *B. fragilis* is resistant to gentamicin and in some cases, gentamicin (20 ug/ml) was added to prevent contamination or counter select *E. coli*. Erythromycin (5 ug/ml) was added to select transposon bearing strains. Solid BHIS plates contained 1.5% agar (Lab Scientific, A466) and 0.001% EDTA. *B. fragilis* manipulations were done aerobically on the benchtop. Incubations were carried out in an anaerobic chamber (Coy Laboratory Products, Grass Lake, MI) filled with 2.5% hydrogen, 97.5% nitrogen. *Escherichia coli* strain AMD776 was grown in LB broth (1% peptone, 0.5% yeast extract, 1% NaCl) supplemented with 100 ug/ml carbenicillin and 0.3 mM diaminopimelic acid (LB-Carb-DAP). *E. coli* was grown shaken aerobically. All incubations were carried out at 37°C unless otherwise noted.

### Dose response of *B. fragilis* P207 to bile salts.

Starter cultures of *B. fragilis* P207 grown overnight in BHIS from freezer stocks were inoculated 1:100 into 2 mL of BHIS supplemented with increasing concentrations of 1) bile salt mixture (50% cholate and 50% deoxycholate; Sigma-Aldrich B8756), 2) deoxycholate (Fisher Scientific BP349), and 3) porcine bile extract (Sigma-Aldrich B8631). Cultures were incubated anaerobically for 24 hours in 14 mm glass tubes. Terminal density of each culture was measured at 600 nm using a Thermo Genesys 20 spectrophotometer. Cells settled at the bottom of the culture were resuspended before measurement.

### B. fragilis P207 genome sequencing.

The complete *B. fragilis* strain P207 genome was produced by first combining published P207 metagenome sequence (24) with long-read sequences collected from an Oxford Nanopore MinIon device. Assembly of long reads plus metagenome sequence was carried out using Flye v2.8 (38) with the metagenome option. Paired-end short reads from an Illumina HiSeq 4000 were then used to polish the Flye assembled genome using Pilon v1.23 (39). This assembly approach yielded a circular genome of 5,040,211 base pairs. The complete genome sequence is available through NCBI GenBank accession CP114371. Reads used to assemble the genome are available at the NCBI Sequence Read Archive (accessions SRR22689962 & SRR22689963.

### Transcriptomic analysis of the B. fragilis P207 bile response.

An overnight starter cultures of B. fragilis P207 was grown from a freezer stock in BHIS at 37°C in a Coy anaerobic chamber. The starters was diluted into triplicate tube with 20 ml fresh BHIS to an OD_600_ of 0.05 and outgrown for 6 hours to an OD_600_ of 0.3. Three ml of each culture was harvested for the untreated control, then 1 ml of a solution containing 0.17% DC in BHIS was added to yield a final concentration of 0.01%. Six minutes after mixing, 3 ml culture was harvested. Again 20 minutes after mixing 3 ml of culture was harvested. Harvesting entailed immediate removal from the anaerobic chamber, centrifugation at 15,000 × g for 1 minute, removal of supernatant and resuspension of the cell pellet in 1 ml TRIzol. Samples were then stored at −80°C until RNA extraction. RNA purification entailed heating the TRIzol samples at 65°C for 10 min followed by addition of 200 μl chloroform, vortexing and incubation at room temperature for 5 min. Aqueous and organic phases were separated by centrifugation at 17,000 × g for 15 min. The upper aqueous phase was transferred to a fresh tube. 0.7X volumes of 100% isopropanol was added, and samples were stored at −80°C overnight. Samples were then centrifuged at 17,000 × g for 30 min at 4°C to pellet the nucleic acid. Pellets were washed twice with 70% ethanol and allowed to dry before resuspension in 100 μl RNase-free water and incubation at 30°C for 10 min. DNAse treatment, stranded library preparation using Illumina’s Stranded Total RNA Prep Ligation with Ribo-Zero Plus kit and custom rRNA depletion probes, and sequencing (2 × 50 bp paired end reads using NextSeq2000) was performed by the Microbial Genome Sequencing Center (Pittsburgh, PA). Reads were mapped to the *B. fragilis* P207 genome (GenBank accession number CP114371) using CLC Genomics Workbench 22 (Qiagen). Differential gene expression False Discovery Rate p-values were calculated using the method of Benjamini and Hochberg (103). A cutoff of absolute transcript log_2_(fold change) ≥ 1.5 and FDR p-value ≤ 10^−10^ was used to determine significant regulation. RNA sequencing reads are available NCBI GEO GSE220692.

### Pathway tools analysis of RNAseq data: assignment of interpro and go terms and pathway enrichment analyses.

Interpro (45) and GO terms (46) to each gene in the *B. fragilis* P207 genome using BioBam Cloud BLAST (Table S2). We then implemented both gene set enrichment analysis (GSEA) (47), and Fisher’s exact test (48) to identify gene function classes that are significantly up- or downregulated after 20 minutes of DC treatment.

### Construction of a pooled barcoded *B. fragilis* P207 Tn-Himar mutant library.

Barcoded himar transposons were introduced into *B. fragilis* P207 from the *E. coli* donor strain, AMD776, carrying the pTGG46-NN1 himar transposon vector library (65) (gift from Adam Deutschbauer, University of California-Berkeley, USA) via conjugation. Briefly, a 10–12 ml BHIS starter culture of *B. fragilis* P207 was inoculated directly from a freezer stock and grown anaerobically for ~16 hours. Simultaneously, 6 ml LB-C-DAP was inoculated with 0.3 ml of a thawed AMD776 pool and shaken for ~16 hours. The *B. fragilis* starter culture was diluted into 150 ml fresh BHIS containing 20 ug/ml gentamicin and grown anaerobically for 6–8 hours while 0.5 ml of the *E. coli* starter was diluted into 100–150 ml of fresh LB-C-DAP and grown aerobically for the same duration. The optical density of each culture was measured at 600nm and the cultures were mixed at approximately 1:1 normalizing by OD. Cells were collected by centrifugation (7,000 g for 4 min) and resuspended in a total of approximately 5 ml fresh BHIS. This cell slurry was transferred in 60–70 spots of 100 ul each to 10–12 BHIS plates containing 0.3 mM diaminopimelic acid to support growth of the *E. coli* donor strain. When the liquid absorbed, the plates were incubated aerobically for ~12 hours and then transferred to the anaerobic incubator for ~12 hours. Groups of 10–12 mating spots were scraped from the plates, resuspended together in 3 ml BHIS and spread evenly over 10–12 BHIS plates containing erythromycin and gentamicin to select for *B. fragilis* harboring the transposon and aid in counter selection of the donor *E. coli* strain. Plates were incubated anaerobically for 2–3 days. Efficiency of transformation was low; approximately 1 × 10^−7^ *B. fragilis* cells became erythromycin resistant and sets of 10–12 conjugation spots would yield 500–5000 transconjugants. Cells from the sets of 10–12 selection plates in each set were scraped and resuspended in 500 ul BHIS then diluted 10-fold to ensure no cell clumps remained. Then a 450 ul aliquot was inoculated into 45 ml BHIS containing erythromycin and gentamicin and outgrown anaerobically for 10–12 hours. Cells in each outgrowth were collected by centrifugation (7000 g for 4 min), resuspended in 20 ml BHIS containing 30% glycerol and stored in aliquots at −80°C. We eventually obtained approximately 70,000 *B. fragilis* P207 strains harboring Tn-himar insertions in 27 small pools. To combine the mutants in each of the smaller pools, each pool was outgrown in parallel and combined proportional to the approximate number of strains in each pool. Specifically, for each pool, 0.3 ml of thawed glycerol stock was inoculated into 13 ml BHIS containing erythromycin and gentamicin and grown anaerobically for 16 hours. After proportional volumes of each pool were mixed together, glycerol was added to a final concentration of 25%. The mixed pool was distributed in 1 ml aliquots and stored at −80°C. Two aliquots were saved for genomic DNA extraction to map transposon insertion sites.

### Mapping Tn-Himar insertion sites in the *B. fragilis* BarSeq library.

Tn insertions sites were mapped following the approach outlined by Wetmore et al. (66) with modifications to the PCR enrichment. Briefly genomic DNA was extracted using guanidium thiocyanate as previously described (104). Four micrograms of genomic DNA in 130 ul volume were sheared using a Covaris M220 ultrasonicator using manufacturers settings to generate ~300 bp fragments. Using the NEBNext Ultra II Library Prep Kit (New England Biolabs, E7103S) following the manufacturers protocol, 1 ug of sheared DNA was end-repaired and A-tailed then ligated to a custom Y adapter, prepared by annealing Mod2_TruSeq and Mod2_TS_Univ (Table S7). The final product was cleaned with SPRIselect beads using a two-sided (0.6–0.25X) selection and eluted from the beads in 25 ul 10 mM Tris, pH 8.5. The fragments containing *Himar* transposons were enriched with a two-step nested PCR strategy using Q5 DNA polymerase with GC enhancer (New England Biolabs, M0491) based on primer sequences (Table S7) originally described by Wetmore et al. (66). In the first step, the forward primer contained the Illumina TruSeq Read 1 region, a random hexamer to facilitate clustering, and a transposon-specific sequence that was extended from the original design to improve specificity, and the reverse primer contained the Illumina TruSeq Read 2 sequence complementary to the adapter. In the second step, the primers result in the addition of Illumina P5 and P7 sequences as well as a 6-bp index on the P7 end. Reaction 1 contained 0.5 uM of each of the primers (TS_bs_T7–35 and TS_R), 0.2 mM dNTP, 1X Q5 reaction buffer, 1X GC enhancer, 2 Units Q5 polymerase and 10 ul of adapter-ligated fragments in a 100 ul reaction volume. Cycling parameters were 98°C for 3 min, 20 × (98°C 30 s, 66°C 20 s, 72°C 20 s), 72°C for 5 min, 4°C hold. The first reaction was cleaned with 0.9X AM-Pure XP beads (Beckman Coulter, A63880) and eluted in 30 ul of 10 mM Tris, pH 8.5. Reaction 2 contained 0.5 uM of each primer (P5_TS_F and P7_MOD_TS_index6), 0.2 mM dNTP, 1X Q5 reaction buffer, 1X GC enhancer, 2 Units Q5 polymerase and 15 ul of cleaned reaction 1 in a 100 ul reaction volume. Cycling parameters were 98°C for 3 min, 15 × (98°C 30 s, 69°C 20 s, 72°C 20 s), 72°C for 5 min, 4°C hold. The final product was cleaned with SPRIselect beads using a two-sided (0.9–0.5X) selection and eluted from the beads in 40 ul 10 mM Tris, pH 8.5. The amplified fragments were sequenced using a 150-cycle MiniSeq High Output Reagent Kit (Illumina, FC-420–1002) on an Illumina MiniSeq. To aid in clustering, sequencing runs were supplemented with 20–30% phiX DNA. Sequences were analyzed using custom scripts written and described by Wetmore and colleagues (66) and available at https://bitbucket.org/berkeleylab/feba/src/master/. Briefly, the locations of Himar transposon insertions were aligned and mapped to the *B. fragilis* P207 chromosome sequence (NCBI accession CP114371) using BLAT, and unique barcode sequences were associated with their corresponding genome insertion location using the custom Perl script, MapTnSeq.pl. Sets of barcodes that reliably map to one location in the genome were identified using the custom Perl script, DesignRandomPool.pl with the following flags -minN 8 – minFrac 0.7 -minRatio 7. Mapping statistics are provided in Table S3. Raw Tn-seq data are deposited in the NCBI sequence read archive under BioProject accession PRJNA910954; BioSample accession SAMN32154224; SRA accession SRR22677646.

### Assessment of *B. fragilis* P207 gene essentiality in BHIS medium.

The *B. fragilis* P207 Tn-himar library was prepared using BHIS growth medium. Quantifying the frequency of insertions across the genome can provide some indication of gene essentiality when cells are grown on BHIS. Analysis of essentiality was performed using the TRANSIT package (available at https://github.com/mad-lab/transit) (105). Briefly, counts of transposon insertions at individual TA dinucleotides sites were measured with the TPP tool in TRANSIT, which uses the BWA aligner (106) to map *himar-B. fragilis* junctional reads to the *B. fragilis* P207 genome. Based on these count data, gene essentiality was calculated using both Gumbel (68) and HMM (67) methods in the TRANSIT package. Output from this analysis is available in Table S5.

### Construction of an arrayed *B. fragilis* P207 Tn-Himar mutant library.

We assembled a limited collection of individual *B. fragilis* P207 *Tn-Himar* mutant strains arrayed in 96-well plates. The mutants were generated by conjugating barcoded transposons from AMD776 into *B. fragilis* P207 as described above. However, instead of pooling transconjugants, individual colonies were manually picked into deep well plates containing 1 ml BHIS with 5 ug/ml erythromycin per well. Plates were incubated anaerobically overnight until wells became turbid. In a fresh deep well plate, 0.5 ml each culture was mixed with 0.5 ml sterile 50% glycerol to yield a final concentration 25% glycerol. The plate with glycerol was sealed with adhesive foil seals and stored at −80°C. A small aliquot from the initial culture plate was saved at −20°C for mapping the transposon insertion sites in each clone (see below). A total of 1020 clones were picked from 2 independent conjugations. This yielded a collection of 889 successfully mapped *B. fragilis* P207∷himar clones representing 723 unique insertion sites. Insertions in 824 clones mapped to predicted coding regions (670 unique sites in 575 unique genes). Thus approximately 14% of genes are represented in this collection. The remaining 65 clones had insertions in predicted non-coding regions. These mapped arrayed mutants are cataloged in Table S4.

### Mapping insertion sites in individual B. fragilis P207 Tn-Himar mutants.

Insertion sites of single clones were mapped using a two-step nested arbitrary PCR approach using 2X GoTaq master mix (Promega M7122). The first reaction (1X GoTaq master mix, 0.3 uM U1 fw, 0.3 uM M13-N7 and 0.5 ul saved culture in a 20 ul reaction) was cycled using the following parameters: 95°C for 2min, 35X (95°C for 30 sec, 38°C for 30 sec, 72°C for 1 min), 72°C for 5 min, 12°C hold. The products were enzymatically cleaned (3 ul of PCR reaction, 2 ul water, 1 ul of ExoSap-IT (Applied Biosystems 78200)) for 15 minutes at 37°C followed by 15 minutes at 80°C. The second PCR reaction (1X GoTaq master mix, 0.3 uM U2 out, 0.3 uM M13Fw and 2 ul of cleaned product above in a 20 ul reaction) was cycled using the following parameters: 95°C for 2 min, 35X (95°C for 30 sec, 68–48°C dropping 0.5°C per cycle for 30 sec, 72°C for 1 min), 72°C for 5 min, 12°C hold. Secondary PCR products were treated with ExoSap-IT as above and Sanger sequenced using the U2 out primer. Sequences were compared to the genome using BLAST to identify insertion sites.

### Cultivation of the Tn-Himar library in bile.

A 0.5 ml aliquot of the pooled *B. fragilis* mutant library glycerol stock was inoculated into 25 ml BHIS containing 5 ug/ml Erythromycin and 20 ug/ml Gentamycin and outgrown anaerobically overnight. Cells from four aliquots of 1 ml each were collected by centrifugation (2 min at 12,000 g), resuspended in 1 ml of phosphate-buffered saline to wash residual media, centrifuged again (2 min at 12,000 g) and stored in pellets −20°C as reference samples. Then 20 ul of overnight culture (containing approximately 2 × 10^8^ CFU) were inoculated into quadruplicate tubes containing 4 mL of either BHIS, BHIS with 0.01% bile salt mix, BHIS with 0.01% deoxycholate. These concentrations reduced growth of wild-type cultures by approximately 20% or 30% respectively (Figure S1). Cultures were incubated anaerobically for 24 h, which allowed for approximately 7 doublings. We harvested cells from 1 ml of these cultures, washing the cell pellets with PBS as we did for the reference samples. In addition, to amplify fitness differences between strains in the mutant pool, we back-diluted these cultures into the same conditions (100 ul into 4 ml fresh media) and allowed a second 24 hour growth period to allow further differentiation of the fitness of individual mutant strains. Again, 1 ml of the passaged day 2 cultures were harvested as above. To assess strain fitness we amplified and sequenced the transposon barcodes after the first and second passages of the pool in the untreated and treatment conditions (see below).

We conducted a similar experiment using porcine bile extract. However, because we experienced more variability in growth with porcine bile extract, we conducted this experiment with three different concentrations, 0.04, 0.08 and 0.16%, using a similar passaging approach from an independent outgrowth of the library. To ensure a maximal number of cell doublings, cultures were incubated until saturation, which was longer than 24 hours at the higher concentrations of porcine bile extract. For the porcine bile extract treatments, barcode abundance was assessed after the second passage.

#### Amplification and sequencing of Tn-Himar barcodes.

To assess barcode abundances, we followed the approach developed and described by Wetmore and colleagues (66). Briefly, each cell pellet was resuspended in approximately 50 ul water. Barcodes were amplified using Q5 polymerase (New England Biolabs) in 20 ul reaction volumes containing 1X Q5 reaction buffer, 1X GC enhancer, 0.8 U Q5 polymerase, 0.2 mM dNTP, 0.5 uM of each primer and 1 ul of resuspended cells. Each reaction contained the Barseq_P1 forward primer and a unique indexed reverse primer, Barseq_P2_ITxxx, where the xxx identifies the index number (66). Reactions were cycled as follows: 98 °C for 4 min, 25X (98 °C for 30 s, 55 °C for 30 s, and 72 °C for 30), 72 °C for 5 min, 4°C hold. PCR products separated on a 2% agarose gel to confirm amplification of a 190 bp product. Aliquots (5 ul) of each reaction were pooled. The pool was centrifuged for 2 min at 16,000g to pellet cell debris. Then 90 ul of the mix was transferred to a fresh tube and cleaned with SPRIselect beads (Beckman Coulter) using a two-sided (1.2x-0.5X) selection. The amplified barcodes were sequenced using a 75-cycle MiniSeq High Output Reagent Kit (Illumina, FC-420–1001) on an Illumina MiniSeq at Michigan State University. To aid in clustering, sequencing runs were supplemented with 20–30% phiX DNA. Sequence data have been deposited in the NCBI Sequence Read Archive under BioProject accession PRJNA910954; BioSample accession SAMN32154224; SRA accession SRR22686218-SRR22686272.

### Analysis of Tn-Himar strain fitness.

Barcode sequences were analyzed using the fitness calculation protocol of Wetmore and colleagues (66). Briefly, the barcodes in each sample were counted and assembled using MultiCodes.pl and combineBarSeq.pl. Then using the barcode abundance data and the mapping information for the mutant pool, gene fitness was calculated using the R script, FEBA.R. The fitness of each strain was calculated as a normalized log_2_ ratio of barcode counts in the treatment sample to counts in the reference sample. The fitness of genes was calculated as the weighted average of strain fitness values, the weight being inversely proportional to a variance metric based on the total number of reads for each strain; this approach is fully described in (66). Insertions in the first 10% or last 10% of a gene were not considered in gene fitness calculations. The complete data set of fitness values and t scores for each condition is listed in Table S6.

To identify genes that contribute to fitness in any bile condition, we filtered the fitness scores in each condition to include scores greater than 1.5 or less than −4 after the second passage. We then manually removed genes which resulted in fitness defects in the absence of bile (average fitness in BHIS < −3). Then 10 genes for which the fitness defect with bile is not substantially worse than without bile (fitness with bile minus fitness defect without bile > −3) were manually removed (PTOS_000072, PTOS_000680, PTOS_001138, PTOS_002708, PTOS_002806, PTOS_003551, PTOS_003802, PTOS_003809, PTOS_003811, PTOS_004091) were removed. This resulted in 122 unique genes whose fitness scores were hierarchically clustered using uncentered correlation and average linkage using Cluster 3.0. Heatmap of clustered fitness scores was rendered with Prism (GraphPad) with some manual rearrangement of genes to bring together genes that are adjacent on the chromosome and have similar fitness profiles.

## Supplementary Material

Supplement 1

Supplement 2

Supplement 3

Supplement 4

Supplement 5

Supplement 6

Supplement 7

1

## References

[1] Kern L, Abdeen SK, Kolodziejczyk AA, Elinav E. 2021. Commensal inter-bacterial interactions shaping the microbiota. Current Opinion in Microbiology 63:158–171. 34365152 10.1016/j.mib.2021.07.011

[2] Keeley TP, Mann GE. 2019. Defining Physiological Normoxia for Improved Translation of Cell Physiology to Animal Models and Humans. Physiological Reviews 99:161–234. 30354965 10.1152/physrev.00041.2017

[3] Fallingborg J, Christensen LA, Ingeman-Nielsen M, Jacobsen BA, Abildgaard K, Rasmussen HH. 1989. pH-profile and regional transit times of the normal gut measured by a radiotelemetry device. Aliment Pharmacol Ther 3:605–13. 2518873 10.1111/j.1365-2036.1989.tb00254.x

[4] Seekatz AM, Schnizlein MK, Koenigsknecht MJ, Baker JR, Hasler WL, Bleske BE, Young VB, Sun D. 2019. Spatial and Temporal Analysis of the Stomach and Small-Intestinal Microbiota in Fasted Healthy Humans. mSphere 4:e00126–19. 30867328 10.1128/mSphere.00126-19 PMC6416366

[5] Guzior DV, Quinn RA. 2021. Review: microbial transformations of human bile acids. Microbiome 9:140. 34127070 10.1186/s40168-021-01101-1 PMC8204491

[6] McNeil NI, Ling KL, Wager J. 1987. Mucosal surface pH of the large intestine of the rat and of normal and inflamed large intestine in man. Gut 28:707–13. 3623217 10.1136/gut.28.6.707 PMC1433035

[7] Bahari HM, Ross IN, Turnberg LA. 1982. Demonstration of a pH gradient across the mucus layer on the surface of human gastric mucosa in vitro. Gut 23:513–6. 7076026 10.1136/gut.23.6.513 PMC1419716

[8] Friedman ES, Bittinger K, Esipova TV, Hou L, Chau L, Jiang J, Mesaros C, Lund PJ, Liang X, FitzGerald GA, Goulian M, Lee D, Garcia BA, Blair IA, Vinogradov SA, Wu GD. 2018. Microbes vs. chemistry in the origin of the anaerobic gut lumen. Proc Natl Acad Sci U S A 115:4170–4175. 29610310 10.1073/pnas.1718635115 PMC5910840

[9] Wiles TJ, Guillemin K. 2019. The other side of the coin: What beneficial microbes can teach us about pathogenic potential. J Mol Biol 431:2946–2956. 31078557 10.1016/j.jmb.2019.05.001

[10] Huang Y, Dalal S, Antonopoulos D, Hubert N, Raffals LH, Dolan K, Weber C, Messer JS, Jabri B, Bendelac A, Eren AM, Rubin DT, Sogin M, Chang EB. 2017. Early Transcriptomic Changes in the Ileal Pouch Provide Insight into the Molecular Pathogenesis of Pouchitis and Ulcerative Colitis. Inflamm Bowel Dis 23:366–378. 28221248 10.1097/MIB.0000000000001027 PMC5988644

[11] Willis S, Kisielinski K, Klosterhalfen B, Schumpelick V. 2002. Morphological and functional adaptation of the small intestine after colectomy and ileal pouch-anal anastomosis in rats. Int J Colorectal Dis 17:85–91. 12014426 10.1007/s003840100352

[12] Apel R, Cohen Z, Andrews CW Jr., McLeod R, Steinhart H, Odze RD. 1994. Prospective evaluation of early morphological changes in pelvic ileal pouches. Gastroenterology 107:435–43. 8039620 10.1016/0016-5085(94)90169-4

[13] Buckman SA, Heise CP. 2010. Nutrition considerations surrounding restorative proctocolectomy. Nutr Clin Pract 25:250–6. 20581318 10.1177/0884533610368708

[14] Nissinen MJ, Gylling H, Järvinen HJ, Miettinen TA. 2004. Ileal pouch-anal anastomosis, conventional ileostomy and ileorectal anastomosis modify cholesterol metabolism. Dig Dis Sci 49:1444–53. 15481317 10.1023/b:ddas.0000042244.56689.72

[15] Hinata M, Kohyama A, Ogawa H, Haneda S, Watanabe K, Suzuki H, Shibata C, Funayama Y, Takahashi K-i, Sasaki I, Fukushima K. 2012. A shift from colon- to ileum-predominant bacteria in ilealpouch feces following total proctocolectomy. Digestive Diseases and Sciences 57:2965–2974. 22539039 10.1007/s10620-012-2165-9

[16] Huse SM, Young VB, Morrison HG, Antonopoulos DA, Kwon J, Dalal S, Arrieta R, Hubert NA, Shen L, Vineis JH, Koval JC, Sogin ML, Chang EB, Raffals LE. 2014. Comparison of brush and biopsy sampling methods of the ileal pouch for assessment of mucosaassociated microbiota of human subjects. Microbiome 2:5. 24529162 10.1186/2049-2618-2-5 PMC3931571

[17] Young VB, Raffals LH, Huse SM, Vital M, Dai D, Schloss PD, Brulc JM, Antonopoulos DA, Arrieta RL, Kwon JH, Reddy KG, Hubert NA, Grim SL, Vineis JH, Dalal S, Morrison HG, Eren AM, Meyer F, Schmidt TM, Tiedje JM, Chang EB, Sogin ML. 2013. Multiphasic analysis of the temporal development of the distal gut microbiota in patients following ileal pouch anal anastomosis. Microbiome 1:9. 24451366 10.1186/2049-2618-1-9 PMC3971607

[18] Kolbeinsson HM, Wall T, Bayat A, Luchtefeld M, Ogilvie JW Jr. 2022. Ileal Pouch Anal Anastomosis (IPAA) for colitis; development of Crohn’s and Pouchitis. Am J Surg 224:453–458. 35086697 10.1016/j.amjsurg.2022.01.018

[19] Sriranganathan D, Kilic Y, Nabil Quraishi M, Segal JP. 2022. Prevalence of pouchitis in both ulcerative colitis and familial adenomatous polyposis: A systematic review and meta-analysis. Colorectal Dis 24:27–39. 34800326 10.1111/codi.15995

[20] Gionchetti P, Calabrese C, Laureti S, Poggioli G, Rizzello F. 2021. Pouchitis: Clinical Features, Diagnosis, and Treatment. Int J Gen Med 14:3871–3879. 34335051 10.2147/IJGM.S306039 PMC8318718

[21] Segal JP, Oke S, Hold GL, Clark SK, Faiz OD, Hart AL. 2018. Systematic review: ileoanal pouch microbiota in health and disease. 47:466–477. 10.1111/apt.14454 29205422

[22] Dubinsky V, Reshef L, Rabinowitz K, Yadgar K, Godny L, Zonensain K, Wasserberg N, Dotan I, Gophna U. 2021. Dysbiosis in Metabolic Genes of the Gut Microbiomes of Patients with an Ileo-anal Pouch Resembles That Observed in Crohn’s Disease. mSystems 6. 10.1128/mSystems.00984-20 PMC8546988 33653942

[23] Gabbiadini R, Dal Buono A, Correale C, Spinelli A, Repici A, Armuzzi A, Roda G. 2022. Ileal Pouch-Anal Anastomosis and Pouchitis: The Role of the Microbiota in the Pathogenesis and Therapy. Nutrients 14. 10.3390/nu14132610 PMC9268595 35807791

[24] Vineis JH, Ringus DL, Morrison HG, Delmont TO, Dalal S, Raffals LH, Antonopoulos DA, Rubin DT, Eren AM, Chang EB, Sogin ML. 2016. Patient-Specific Bacteroides Genome Variants in Pouchitis. mBio 7. 10.1128/mBio.01713-16 PMC5111406 27935837

[25] Floch MH, Binder HJ, Filburn B, Gershengoren W. 1972. The effect of bile acids on intestinal microflora. Am J Clin Nutr 25:1418–26. 4344803 10.1093/ajcn/25.12.1418

[26] Begley M, Gahan CGM, Hill C. 2005. The interaction between bacteria and bile. FEMS Microbiology Reviews 29:625–651. 16102595 10.1016/j.femsre.2004.09.003

[27] Kurdi P, Kawanishi K, Mizutani K, Yokota A. 2006. Mechanism of growth inhibition by free bile acids in lactobacilli and bifidobacteria. J Bacteriol 188:1979–86. 16484210 10.1128/JB.188.5.1979-1986.2006 PMC1426545

[28] Merritt ME, Donaldson JR. 2009. Effect of bile salts on the DNA and membrane integrity of enteric bacteria. J Med Microbiol 58:1533–1541. 19762477 10.1099/jmm.0.014092-0

[29] Islam KB, Fukiya S, Hagio M, Fujii N, Ishizuka S, Ooka T, Ogura Y, Hayashi T, Yokota A. 2011. Bile acid is a host factor that regulates the composition of the cecal microbiota in rats. Gastroenterology 141:1773–81. 21839040 10.1053/j.gastro.2011.07.046

[30] Long SL, Gahan CGM, Joyce SA. 2017. Interactions between gut bacteria and bile in health and disease. Mol Aspects Med 56:54–65. 28602676 10.1016/j.mam.2017.06.002

[31] Sievers S, Metzendorf NG, Dittmann S, Troitzsch D, Gast V, Troger SM, Wolff C, Zuhlke D, Hirschfeld C, Schluter R, Riedel K. 2019. Differential View on the Bile Acid Stress Response of Clostridioides difficile. Front Microbiol 10:258. 30833939 10.3389/fmicb.2019.00258 PMC6387971

[32] Larabi AB, Masson HLP, Baumler AJ. 2023. Bile acids as modulators of gut microbiota composition and function. Gut Microbes 15:2172671. 36740850 10.1080/19490976.2023.2172671 PMC9904317

[33] Theriot CM, Bowman AA, Young VB. 2016. Antibiotic-Induced Alterations of the Gut Microbiota Alter Secondary Bile Acid Production and Allow for Clostridium difficile Spore Germination and Outgrowth in the Large Intestine. mSphere 1. 10.1128/mSphere.00045-15 PMC4863611 27239562

[34] Bustos AY, Font de Valdez G, Fadda S, Taranto MP. 2018. New insights into bacterial bile resistance mechanisms: the role of bile salt hydrolase and its impact on human health. Food Research International 112:250–262. 30131136 10.1016/j.foodres.2018.06.035

[35] Sinha SR, Haileselassie Y, Nguyen LP, Tropini C, Wang M, Becker LS, Sim D, Jarr K, Spear ET, Singh G, Namkoong H, Bittinger K, Fischbach MA, Sonnenburg JL, Habtezion A. 2020. Dysbiosis Induced Secondary Bile Acid Deficiency Promotes Intestinal Inflammation. Cell Host Microbe 27:659–670.e5. 32101703 10.1016/j.chom.2020.01.021 PMC8172352

[36] Livingston SJ, Kominos SD, Yee RB. 1978. New medium for selection and presumptive identification of the Bacteroides fragilis group. J Clin Microbiol 7:448–53. 659574 10.1128/jcm.7.5.448-453.1978 PMC275014

[37] Draper DL, Barry AL. 1977. Rapid identification of Bacteroides fragilis with bile and antibiotic disks. J Clin Microbiol 5:439–43. 323285 10.1128/jcm.5.4.439-443.1977 PMC274620

[38] Kolmogorov M, Yuan J, Lin Y, Pevzner PA. 2019. Assembly of long, error-prone reads using repeat graphs. Nat Biotechnol 37:540–546. 30936562 10.1038/s41587-019-0072-8

[39] Walker BJ, Abeel T, Shea T, Priest M, Abouelliel A, Sakthikumar S, Cuomo CA, Zeng Q, Wortman J, Young SK, Earl AM. 2014. Pilon: an integrated tool for comprehensive microbial variant detection and genome assembly improvement. PLoS One 9:e112963. 25409509 10.1371/journal.pone.0112963 PMC4237348

[40] Tatusova T, DiCuccio M, Badretdin A, Chetvernin V, Nawrocki EP, Zaslavsky L, Lomsadze A, Pruitt KD, Borodovsky M, Ostell J. 2016. NCBI prokaryotic genome annotation pipeline. Nucleic Acids Res 44:6614–24. 27342282 10.1093/nar/gkw569 PMC5001611

[41] Franco AA, Cheng RK, Chung GT, Wu S, Oh HB, Sears CL. 1999. Molecular evolution of the pathogenicity island of enterotoxigenic Bacteroides fragilis strains. J Bacteriol 181:6623–33. 10542162 10.1128/jb.181.21.6623-6633.1999 PMC94125

[42] Kato N, Liu CX, Kato H, Watanabe K, Tanaka Y, Yamamoto T, Suzuki K, Ueno K. 2000. A new subtype of the metalloprotease toxin gene and the incidence of the three bft subtypes among Bacteroides fragilis isolates in Japan. FEMS Microbiol Lett 182:171–6. 10612750 10.1111/j.1574-6968.2000.tb08892.x

[43] Franco AA, Mundy LM, Trucksis M, Wu S, Kaper JB, Sears CL. 1997. Cloning and characterization of the Bacteroides fragilis metalloprotease toxin gene. Infect Immun 65:1007–13. 9038310 10.1128/iai.65.3.1007-1013.1997 PMC175082

[44] Chung GT, Franco AA, Wu S, Rhie GE, Cheng R, Oh HB, Sears CL. 1999. Identification of a third metalloprotease toxin gene in extraintestinal isolates of Bacteroides fragilis. Infect Immun 67:4945–9. 10456956 10.1128/iai.67.9.4945-4949.1999 PMC96834

[45] Blum M, Chang HY, Chuguransky S, Grego T, Kandasaamy S, Mitchell A, Nuka G, Paysan-Lafosse T, Qureshi M, Raj S, Richardson L, Salazar GA, Williams L, Bork P, Bridge A, Gough J, Haft DH, Letunic I, Marchler-Bauer A, Mi H, Natale DA, Necci M, Orengo CA, Pandurangan AP, Rivoire C, Sigrist CJA, Sillitoe I, Thanki N, Thomas PD, Tosatto SCE, Wu CH, Bateman A, Finn RD. 2021. The InterPro protein families and domains database: 20 years on. Nucleic Acids Res 49:D344–D354. 33156333 10.1093/nar/gkaa977 PMC7778928

[46] Gene Ontology C. 2021. The Gene Ontology resource: enriching a GOld mine. Nucleic Acids Res 49:D325–D334. 33290552 10.1093/nar/gkaa1113 PMC7779012

[47] Subramanian A, Tamayo P, Mootha VK, Mukherjee S, Ebert BL, Gillette MA, Paulovich A, Pomeroy SL, Golub TR, Lander ES, Mesirov JP. 2005. Gene set enrichment analysis: a knowledge-based approach for interpreting genome-wide expression profiles. Proc Natl Acad Sci U S A 102:15545–50. 16199517 10.1073/pnas.0506580102 PMC1239896

[48] Al-Shahrour F, Diaz-Uriarte R, Dopazo J. 2004. FatiGO: a web tool for finding significant associations of Gene Ontology terms with groups of genes. Bioinformatics 20:578–80. 14990455 10.1093/bioinformatics/btg455

[49] Ulrich LE, Koonin EV, Zhulin IB. 2005. One-component systems dominate signal transduction in prokaryotes. Trends Microbiol 13:52–6. 15680762 10.1016/j.tim.2004.12.006 PMC2756188

[50] Stock AM, Robinson VL, Goudreau PN. 2000. Two-component signal transduction. Annu Rev Biochem 69:183–215. 10966457 10.1146/annurev.biochem.69.1.183

[51] Alcantara C, Blasco A, Zuniga M, Monedero V. 2014. Accumulation of polyphosphate in Lactobacillus spp. and its involvement in stress resistance. Appl Environ Microbiol 80:1650–9. 24375133 10.1128/AEM.03997-13 PMC3957611

[52] Gray MJ, Wholey WY, Wagner NO, Cremers CM, Mueller-Schickert A, Hock NT, Krieger AG, Smith EM, Bender RA, Bardwell JC, Jakob U. 2014. Polyphosphate is a primordial chaperone. Mol Cell 53:689–99. 24560923 10.1016/j.molcel.2014.01.012 PMC3996911

[53] Henry JT, Crosson S. 2013. Chromosome replication and segregation govern the biogenesis and inheritance of inorganic polyphosphate granules. Mol Biol Cell 24:3177–86. 23985321 10.1091/mbc.E13-04-0182 PMC3806658

[54] Rao NN, Gomez-Garcia MR, Kornberg A. 2009. Inorganic polyphosphate: essential for growth and survival. Annu Rev Biochem 78:605–47. 19344251 10.1146/annurev.biochem.77.083007.093039

[55] Boutte CC, Crosson S. 2013. Bacterial lifestyle shapes stringent response activation. Trends Microbiol 21:174–80. 23419217 10.1016/j.tim.2013.01.002 PMC4238387

[56] Feehily C, Karatzas KA. 2013. Role of glutamate metabolism in bacterial responses towards acid and other stresses. J Appl Microbiol 114:11–24. 22924898 10.1111/j.1365-2672.2012.05434.x

[57] Otaru N, Ye K, Mujezinovic D, Berchtold L, Constancias F, Cornejo FA, Krzystek A, de Wouters T, Braegger C, Lacroix C, Pugin B. 2021. GABA Production by Human Intestinal Bacteroides spp.: Prevalence, Regulation, and Role in Acid Stress Tolerance. Front Microbiol 12:656895. 33936013 10.3389/fmicb.2021.656895 PMC8082179

[58] Bron PA, Molenaar D, de Vos WM, Kleerebezem M. 2006. DNA micro-array-based identification of bile-responsive genes in Lactobacillus plantarum. J Appl Microbiol 100:728–38. 16553727 10.1111/j.1365-2672.2006.02891.x

[59] Begley M, Gahan CG, Hill C. 2002. Bile stress response in Listeria monocytogenes LO28: adaptation, cross-protection, and identification of genetic loci involved in bile resistance. Appl Environ Microbiol 68:6005–12. 12450822 10.1128/AEM.68.12.6005-6012.2002 PMC134417

[60] Luoto HH, Baykov AA, Lahti R, Malinen AM. 2013. Membrane-integral pyrophosphatase subfamily capable of translocating both Na+ and H+. Proc Natl Acad Sci U S A 110:1255–60. 23297210 10.1073/pnas.1217816110 PMC3557053

[61] Luoto HH, Nordbo E, Baykov AA, Lahti R, Malinen AM. 2013. Membrane Na+-pyrophosphatases can transport protons at low sodium concentrations. J Biol Chem 288:35489–99. 24158447 10.1074/jbc.M113.510909 PMC3853295

[62] Du D, Wang-Kan X, Neuberger A, van Veen HW, Pos KM, Piddock LJV, Luisi BF. 2018. Multidrug efflux pumps: structure, function and regulation. Nat Rev Microbiol 16:523–539. 30002505 10.1038/s41579-018-0048-6

[63] Koronakis V. 2003. TolC--the bacterial exit duct for proteins and drugs. FEBS Lett 555:66–71. 14630321 10.1016/s0014-5793(03)01125-6

[64] Pumbwe L, Skilbeck CA, Nakano V, Avila-Campos MJ, Piazza RM, Wexler HM. 2007. Bile salts enhance bacterial co-aggregation, bacterial-intestinal epithelial cell adhesion, biofilm formation and antimicrobial resistance of Bacteroides fragilis. Microb Pathog 43:78–87. 17524609 10.1016/j.micpath.2007.04.002

[65] Liu H, Shiver AL, Price MN, Carlson HK, Trotter VV, Chen Y, Escalante V, Ray J, Hern KE, Petzold CJ, Turnbaugh PJ, Huang KC, Arkin AP, Deutschbauer AM. 2021. Functional genetics of human gut commensal Bacteroides thetaiotaomicron reveals metabolic requirements for growth across environments. Cell Rep 34:108789. 33657378 10.1016/j.celrep.2021.108789 PMC8121099

[66] Wetmore KM, Price MN, Waters RJ, Lamson JS, He J, Hoover CA, Blow MJ, Bristow J, Butland G, Arkin AP, Deutschbauer A. 2015. Rapid quantification of mutant fitness in diverse bacteria by sequencing randomly bar-coded transposons. mBio 6:e00306–15. 25968644 10.1128/mBio.00306-15 PMC4436071

[67] DeJesus MA, Ioerger TR. 2013. A Hidden Markov Model for identifying essential and growth-defect regions in bacterial genomes from transposon insertion sequencing data. BMC Bioinformatics 14:303. 24103077 10.1186/1471-2105-14-303 PMC3854130

[68] DeJesus MA, Zhang YJ, Sassetti CM, Rubin EJ, Sacchettini JC, Ioerger TR. 2013. Bayesian analysis of gene essentiality based on sequencing of transposon insertion libraries. Bioinformatics 29:695–703. 23361328 10.1093/bioinformatics/btt043 PMC3597147

[69] Schofield WB, Zimmermann-Kogadeeva M, Zimmermann M, Barry NA, Goodman AL. 2018. The Stringent Response Determines the Ability of a Commensal Bacterium to Survive Starvation and to Persist in the Gut. Cell Host Microbe 24:120–132 e6. 30008292 10.1016/j.chom.2018.06.002 PMC6086485

[70] Brigham CJ, Malamy MH. 2005. Characterization of the RokA and HexA broad-substrate-specificity hexokinases from Bacteroides fragilis and their role in hexose and N-acetylglucosamine utilization. J Bacteriol 187:890–901. 15659667 10.1128/JB.187.3.890-901.2005 PMC545704

[71] Veeranagouda Y, Husain F, Tenorio EL, Wexler HM. 2014. Identification of genes required for the survival of B. fragilis using massive parallel sequencing of a saturated transposon mutant library. BMC Genomics 15:429. 24899126 10.1186/1471-2164-15-429 PMC4072883

[72] Chatzidaki-Livanis M, Weinacht KG, Comstock LE. 2010. Trans locus inhibitors limit concomitant polysaccharide synthesis in the human gut symbiont Bacteroides fragilis. Proc Natl Acad Sci U S A 107:11976–80. 20547868 10.1073/pnas.1005039107 PMC2900635

[73] Chatzidaki-Livanis M, Coyne MJ, Roche-Hakansson H, Comstock LE. 2008. Expression of a uniquely regulated extracellular polysaccharide confers a large-capsule phenotype to Bacteroides fragilis. J Bacteriol 190:1020–6. 18039760 10.1128/JB.01519-07 PMC2223589

[74] Romantsov T, Guan Z, Wood JM. 2009. Cardiolipin and the osmotic stress responses of bacteria. Biochim Biophys Acta 1788:2092–100. 19539601 10.1016/j.bbamem.2009.06.010 PMC3622477

[75] Kato S, Tobe H, Matsubara H, Sawada M, Sasaki Y, Fukiya S, Morita N, Yokota A. 2019. The membrane phospholipid cardiolipin plays a pivotal role in bile acid adaptation by Lactobacillus gasseri JCM1131(T). Biochim Biophys Acta Mol Cell Biol Lipids 1864:403–412. 29883797 10.1016/j.bbalip.2018.06.004

[76] Zhang X, Bierschenk D, Top J, Anastasiou I, Bonten MJ, Willems RJ, van Schaik W. 2013. Functional genomic analysis of bile salt resistance in Enterococcus faecium. BMC Genomics 14:299. 23641968 10.1186/1471-2164-14-299 PMC3653699

[77] Coyne MJ, Fletcher CM, Chatzidaki-Livanis M, Posch G, Schaffer C, Comstock LE. 2013. Phylum-wide general protein O-glycosylation system of the Bacteroidetes. Mol Microbiol 88:772–83. 23551589 10.1111/mmi.12220 PMC3656502

[78] Coyne MJ, Reinap B, Lee MM, Comstock LE. 2005. Human symbionts use a host-like pathway for surface fucosylation. Science 307:1778–81. 15774760 10.1126/science.1106469

[79] Fletcher CM, Coyne MJ, Villa OF, Chatzidaki-Livanis M, Comstock LE. 2009. A general O-glycosylation system important to the physiology of a major human intestinal symbiont. Cell 137:321–31. 19379697 10.1016/j.cell.2009.02.041 PMC2772059

[80] Fletcher CM, Coyne MJ, Comstock LE. 2011. Theoretical and experimental characterization of the scope of protein O-glycosylation in Bacteroides fragilis. J Biol Chem 286:3219–26. 21115495 10.1074/jbc.M110.194506 PMC3030326

[81] Sijbrandi R, Stork M, Luirink J, Otto BR. 2008. Pbp, a cell-surface exposed plasminogen binding protein of Bacteroides fragilis. Microbes Infect 10:514–21. 18403231 10.1016/j.micinf.2008.01.015

[82] Wilson MM, Anderson DE, Bernstein HD. 2015. Analysis of the outer membrane proteome and secretome of Bacteroides fragilis reveals a multiplicity of secretion mechanisms. PLoS One 10:e0117732. 25658944 10.1371/journal.pone.0117732 PMC4319957

[83] Mulkidjanian AY, Dibrov P, Galperin MY. 2008. The past and present of sodium energetics: may the sodium-motive force be with you. Biochim Biophys Acta 1777:985–92. 18485887 10.1016/j.bbabio.2008.04.028 PMC2695506

[84] Meier T, Polzer P, Diederichs K, Welte W, Dimroth P. 2005. Structure of the rotor ring of F-Type Na+-ATPase from Ilyobacter tartaricus. Science 308:659–62. 15860619 10.1126/science.1111199

[85] Murata T, Yamato I, Kakinuma Y, Leslie AG, Walker JE. 2005. Structure of the rotor of the V-Type Na+-ATPase from Enterococcus hirae. Science 308:654–9. 15802565 10.1126/science.1110064

[86] Tang YP, Dallas MM, Malamy MH. 1999. Characterization of the Batl (Bacteroides aerotolerance) operon in Bacteroides fragilis: isolation of a B. fragilis mutant with reduced aerotolerance and impaired growth in in vivo model systems. Mol Microbiol 32:139–49. 10216867 10.1046/j.1365-2958.1999.01337.x

[87] Anes J, McCusker MP, Fanning S, Martins M. 2015. The ins and outs of RND efflux pumps in Escherichia coli. Front Microbiol 6:587. 26113845 10.3389/fmicb.2015.00587 PMC4462101

[88] Arias-Cartin R, Grimaldi S, Arnoux P, Guigliarelli B, Magalon A. 2012. Cardiolipin binding in bacterial respiratory complexes: structural and functional implications. Biochim Biophys Acta 1817:1937–49. 22561115 10.1016/j.bbabio.2012.04.005

[89] Mehdipour AR, Hummer G. 2016. Cardiolipin puts the seal on ATP synthase. Proc Natl Acad Sci U S A 113:8568–70. 27439859 10.1073/pnas.1609806113 PMC4978257

[90] Mileykovskaya E, Dowhan W. 2009. Cardiolipin membrane domains in prokaryotes and eukaryotes. Biochim Biophys Acta 1788:2084–91. 19371718 10.1016/j.bbamem.2009.04.003 PMC2757463

[91] Musatov A, Sedlak E. 2017. Role of cardiolipin in stability of integral membrane proteins. Biochimie 142:102–111. 28842204 10.1016/j.biochi.2017.08.013

[92] Zhou M, Morgner N, Barrera NP, Politis A, Isaacson SC, Matak-Vinkovic D, Murata T, Bernal RA, Stock D, Robinson CV. 2011. Mass spectrometry of intact V-type ATPases reveals bound lipids and the effects of nucleotide binding. Science 334:380–385. 22021858 10.1126/science.1210148 PMC3927129

[93] Duncan AL, Robinson AJ, Walker JE. 2016. Cardiolipin binds selectively but transiently to conserved lysine residues in the rotor of metazoan ATP synthases. Proc Natl Acad Sci U S A 113:8687–92. 27382158 10.1073/pnas.1608396113 PMC4978264

[94] Gupta K, Donlan JAC, Hopper JTS, Uzdavinys P, Landreh M, Struwe WB, Drew D, Baldwin AJ, Stansfeld PJ, Robinson CV. 2017. The role of interfacial lipids in stabilizing membrane protein oligomers. Nature 541:421–424. 28077870 10.1038/nature20820 PMC5501331

[95] Haines TH, Dencher NA. 2002. Cardiolipin: a proton trap for oxidative phosphorylation. FEBS Lett 528:35–9. 12297275 10.1016/s0014-5793(02)03292-1

[96] Lohmeier-Vogel EM, Leung KT, Lee H, Trevors JT, Vogel HJ. 2001. Phosphorus-31 nuclear magnetic resonance study of the effect of pentachlorophenol (PCP) on the physiologies of PCP-degrading microorganisms. Appl Environ Microbiol 67:3549–56. 11472931 10.1128/AEM.67.8.3549-3556.2001 PMC93055

[97] Nakano M, Imamura H, Toei M, Tamakoshi M, Yoshida M, Yokoyama K. 2008. ATP hydrolysis and synthesis of a rotary motor V-ATPase from Thermus thermophilus. J Biol Chem 283:20789–96. 18492667 10.1074/jbc.M801276200 PMC3258951

[98] Kakinuma Y, Yamato I, Murata T. 1999. Structure and function of vacuolar Na+-translocating ATPase in Enterococcus hirae. J Bioenerg Biomembr 31:7–14. 10340844 10.1023/a:1005499126939

[99] Fischer CL, Dawson DV, Blanchette DR, Drake DR, Wertz PW, Brogden KA. 2016. Protein Analysis of Sapienic Acid-Treated Porphyromonas gingivalis Suggests Differential Regulation of Multiple Metabolic Pathways. J Bacteriol 198:157–67. 26483519 10.1128/JB.00665-15 PMC4686187

[100] Ito T, Gallegos R, Matano LM, Butler NL, Hantman N, Kaili M, Coyne MJ, Comstock LE, Malamy MH, Barquera B. 2020. Genetic and Biochemical Analysis of Anaerobic Respiration in Bacteroides fragilis and Its Importance In Vivo. mBio 11. 10.1128/mBio.03238-19 PMC7002350 32019804

[101] Barkas F, Liberopoulos E, Kei A, Elisaf M. 2013. Electrolyte and acid-base disorders in inflammatory bowel disease. Ann Gastroenterol 26:23–28. 24714322 PMC3959504

[102] Lolkema JS, Chaban Y, Boekema EJ. 2003. Subunit composition, structure, and distribution of bacterial V-type ATPases. J Bioenerg Biomembr 35:323–35. 14635778 10.1023/a:1025776831494

[103] Benjamini Y, Hochberg Y. 1995. Controlling the False Discovery Rate: A Practical and Powerful Approach to Multiple Testing. Journal of the Royal Statistical Society: Series B (Methodological) 57:289–300.

[104] Pitcher DG, Saunders NA, Owen RJ. 1989. Rapid extraction of bacterial genomic DNA with guanidium thiocyanate. Lett Appl Microbiol 8:151–156.

[105] DeJesus MA, Ambadipudi C, Baker R, Sassetti C, Ioerger TR. 2015. TRANSIT--A Software Tool for Himar1 TnSeq Analysis. PLoS Comput Biol 11:e1004401. 26447887 10.1371/journal.pcbi.1004401 PMC4598096

[106] Li H, Durbin R. 2009. Fast and accurate short read alignment with Burrows-Wheeler transform. Bioinformatics 25:1754–60. 19451168 10.1093/bioinformatics/btp324 PMC2705234

[107] Ueno H, Suzuki K, Murata T. 2018. Structure and dynamics of rotary V(1) motor. Cell Mol Life Sci 75:1789–1802. 29387903 10.1007/s00018-018-2758-3 PMC5910484

