# Supplementary material for: Bile acid fitness determinants of a Bacteroides fragilis isolate from a human pouchitis patient
Source: bioRxiv. 2023 Oct 17:2023.05.11.540287. Originally published 2023 May 11. Preprint. [Version 2] doi: 10.1101/2023.05.11.540287 (PMC10197588; doi:10.1101/2023.05.11.540287)
Supplement: Supplement 7 [file media-7.docx]

**Table S7: Strains and primers**

| **Strain** | **Genotype (description)** | **Source** |
| --- | --- | --- |
| AMD776 | E. coli WM3064 / pTGG46_NN1  (Donor strain carrying barcoded ErmR TnHimar containing plasmid) | (1) |
| FC3835 | Bacteroides fragilis str. P207  (Isolate from pouchitis patient with inflammation) | (2) |
|  | B. fragilis str P207 :: pTGG46_NN1  (mixed pool of barcoded TnHimar mutants) | This study |
| **Primer / oligo** | **5’-3’ Sequence (description)** | **Notes** |
| Y adapter oligos | | |
| Mod2_TruSeq | /5'P/GATCGGAAGAGCACACGTCTGAACTCCAGTCA  (5’ phosphorylation) | Y-adapter (3) |
| Mod2_TS_Univ | ACGCTCTTCCGATC*T  (3' T is phosphorothioate bonded) | Y-adapter (3) |
| For two-step nested amplification of barcode junctions - TNseq | | |
| TS_bs_T7-35 | ACACTCTTTCCCTACACGACGCTCTTCCGATCTNNNNNNCGACTCACTATAGGGGATA**GATGTCCACGAG**GTCT  (TruSeqRead1-hexamer-TN specific) | Modified from Nspacer_barseq_universal (3) to increase transposon specific sequence |
| TS_R | GTGACTGGAGTTCAGACGTGTGCTCTTCCGATCT  (TruSeqRead2) |  |
| P5-TS F | AATGATACGGCGACCACCGAGATCTACACTCTTTCCCTACACGACGCTCTTCCGATCT  (P5 sequence-TruSeqRead1) |  |
| P7_MOD_TS_index6 | CAAGCAGAAGACGGCATACGAGAT**CGTGAT**GTGACTGGAGTTCAGACGTGTGCTCTTCCGATCT  (P7 sequence-**index**-TruSeqRead2) | (3) |
| For amplification of barcodes - BarSeq | | |
| Barseq_P1 | AATGATACGGCGACCACCGAGATCTACACTCTTTCCCTACACGACGCTCTTCCGATCTNNNNNGTCGACCTGCAGCGTACG  (P5-TruSeqRead1-hexamer-TN specific) | (3) |
| Barseq_P2_ITxxx | CAAGCAGAAGACGGCATACGAGATXXXXXXGTGACTGGAGTTCAGACGTGTGCTCTTCCGATCTGATGTCCACGAGGTCTCT  (P7 sequence-index-TruSeqRead2-TN specific) | Uniquely indexed primers were used for each sample and are fully listed in (3) |
| For arbitrary nested amplification of barcode junctions in single clones | | |
| U1 F | GATGTCCACGAGGTCTCT |  |
| M13F-N_7_ | TGTAAAACGACGGCCAGTNNNNNNN  (M13F-random heptamer) |  |
| U2 out | CGTACGCTGCAGGTCGAC |  |
| M13F | TGTAAAACGACGGCCAGT |  |
| **For RT-qPCR** | | |
| PTOS_001192_F | GCATCGGGTTGCTAATCGTT | Hypothetical |
| PTOS_001192_R | AGAAGCATCACGAGCCAACA |  |
| PTOS_001365_F | ACACCTCTCCCACCCCAATA | DEAD/DEAH box helicase |
| PTOS_001365_R | GCAGCTGTTTTACCGGTACCT |  |
| PTOS_001272_F | CGAAATTGCAAAGAACACCGGT | Integration host factor |
| PTOS_001272_R | CGAAGCTACCAAATCCACGG |  |
| PTOS_003612_F | CTTGCATTCCTCACCCTGCT | RND efflux transporter |
| PTOS_003612_R | TTCGGAGCTGCGATGACTAC |  |
| PTOS_001049_F | TTGCCCTTGCCCTCTTTTCT | DnaK |
| PTOS_001049_R | CACAGCCACCATAGTAGCGT |  |
| PTOS_002119_F | GGCATTGAGTTGATCGCATCA | Superoxide dismutase |
| PTOS_002119_R | AGTACTGCACTTTTCAGCCA |  |
| PTOS_001852_F | GGCATTGAGTTGATCGCATCA | Serine hydroxymethyltransferase |
| PTOS_001852_R | CACAGCCACCATAGTAGCGT |  |
| PTOS_001202_F | ACAGTGTTCCGTCCTCCAAC | σ^70^ RNA polymerase sigma factor |
| PTOS_001202_R | GCATCGGGTTGCTAATCGTT |  |

**References:**

1. Liu H, Shiver AL, Price MN, Carlson HK, Trotter VV, Chen Y, Escalante V, Ray J, Hern KE, Petzold CJ, Turnbaugh PJ, Huang KC, Arkin AP, Deutschbauer AM. 2021. Functional genetics of human gut commensal Bacteroides thetaiotaomicron reveals metabolic requirements for growth across environments. Cell Rep 34:108789.

2. Vineis JH, Ringus DL, Morrison HG, Delmont TO, Dalal S, Raffals LH, Antonopoulos DA, Rubin DT, Eren AM, Chang EB, Sogin ML. 2016. Patient-Specific Bacteroides Genome Variants in Pouchitis. mBio 7.

3. Wetmore KM, Price MN, Waters RJ, Lamson JS, He J, Hoover CA, Blow MJ, Bristow J, Butland G, Arkin AP, Deutschbauer A. 2015. Rapid quantification of mutant fitness in diverse bacteria by sequencing randomly bar-coded transposons. mBio 6:e00306-15.
