# Supplementary material for: Bile acid fitness determinants of a Bacteroides fragilis isolate from a human pouchitis patient
Source: bioRxiv. 2023 Oct 17:2023.05.11.540287. Originally published 2023 May 11. Preprint. [Version 2] doi: 10.1101/2023.05.11.540287 (PMC10197588; doi:10.1101/2023.05.11.540287)
Supplement: Supplement 8 [file NIHPP2023.05.11.540287v2-supplement-8.pdf]

bioRxiv preprint doi: https://doi.org/10.1101/2023.05.11.540287; this version posted October 17, 2023. The copyright holder for this preprint (which was not certified by peer review) is the author/funder, who has granted bioRxiv a license to display the preprint in perpetuity. It is made available under aCC-BY 4.0 International license.

Supplemental Materials

1) Supplemental Results

Multiple classes of transcription factors are regulated by DC
Transcriptional evidence for a metabolic shift upon DC exposure
DC-regulated expression of membrane ion transport systems
B. fragilis P207 relies on multiple efflux and stress response systems in the presence of bile
Candidate Essential Genes of Note in B. fragilis P207

2) Supplemental Figures

Figure S1: Representative growth curves of *B. fragilis P207* grown in BHIS in the presence of increasing concentrations of bile
Figure S2: Evaluation of changes in transcript abundance 20 minutes after deoxycholate treatment by RT-qPCR
Figure S3: BarSeq replicate data are consistent within each treatment group
Figure S4: Individual transposon mutant strains validate fitness phenotypes observed in mutant pool
Figure S5: Transcriptionally regulated genes with significant fitness scores
Figure S6: Transcriptional regulators exhibit a complex response to DC.
Figure S7: Multiple efflux systems differentially contribute to *B. fragilis* fitness in the presence of purified bile acid and crude bile conditions.
Figure S8: Expression and fitness scores for all 23 TolC-family membrane transport systems.
Figure S9: A broad repertoire of stress response systems enable survival in deoxycholate

In separate documents:

Table S1: RNAseq expression data.xlsx
Table S2: Overrepresented_functional_groups_in_up_and_downregulated_genes.xlsx
Table S3: Mapping statistics
Table S4: P207-TN_arrayed_collection.xlsx
Table S5: Essential gene calls.xlsx
Table S6: BarSeq output.xlsx
Table S7: Strains and primers

bioRxiv preprint doi: https://doi.org/10.1101/2023.05.11.540287; this version posted October 17, 2023. The copyright holder for this preprint (which was not certified by peer review) is the author/funder, who has granted bioRxiv a license to display the preprint in perpetuity. It is made available under aCC-BY 4.0 International license.

Supplemental Results

Multiple classes of transcription factors are regulated by DC

Approximately one quarter of all *B. fragilis* P207 genes exhibited significant differential expression upon acute DC exposure. The massive transcriptional response that we observe upon DC treatment is likely a result of a cascade of transcriptional regulatory events cued by stress inflicted by deoxycholate. Two of the top three enriched functional categories in the activated gene set relate to transcription (enriched GO terms: transcription cis-regulatory region binding, and regulation DNA-templated transcription; Table S2). The *B. fragilis* sigma factors had a striking profile in which eight sigma factors were activated and ten repressed ($|\log_2(FC)|>1.5$) by the 20 minute time point, (Figure S6A; Tables S1 & S2). Other common classes of transcriptional regulators included in the enriched gene set include one-component (1) and two-component system (2) genes, which show a similarly disparate regulatory profile as the alternative sigma factors (Figure S6 B-C; Tables S1 & S2), indicating a complex response to DC exposure.

Transcriptional evidence for a metabolic shift upon DC exposure

By far, the most significantly enriched functional categories in the down-regulated set of genes involve translation (enriched GO terms: ribosome, translational elongation, aminoacyl-tRNA ligase activity, tRNA aminoacylation for protein translation). Expression of ribosomal proteins, ribosomal accessory factors, and aminoacyl tRNA synthetases is uniformly lower (by 2-50 fold), with few exceptions (main text Figure 1B-C, Tables S1 & S2). We note that most of these genes are not represented in our fitness data as translation is an essential process. The transcriptional effect observed resembles gene expression changes induced by the stringent response (3), which slows growth and facilitates cellular adaptation to nutrient or energy limitation. The wholesale downregulation of protein expression machinery is accompanied by reduced expression of genes required for cell growth and division (enriched GO terms: regulation of cell shape, cell wall organization, peptidoglycan biosynthetic process, lipopolysaccharide biosynthetic process, cell envelope) (Tables S1 & S2). Other significantly down-regulated biosynthetic pathways (FDR q-value < 0.05) include genes required for production of cobalamin, ribonucleosides, pseudouridine, pantothenic acid, queuosine, arginine, and coenzyme A.

While the transcriptomic data provide evidence that deoxycholate treatment results in a large-scale downregulation of anabolic metabolism, expression of select metabolic enzymes is activated. Among the most notable of these responses is the transcriptional activation of genes involved in glutamate metabolism (enriched GO term: glutamate metabolic process) (Table S2). Specifically, DC treatment induces expression of asparagine synthase B (*asnB; ptos_002403*), glutaminase (*glsA; ptos_000368*), genes in the histidine utilization pathway (*ptos_003734-3738*), and glutamate dehydrogenase (*gdhA; ptos_003163*), all of which catalyze production of glutamate from various substrates. The expression of glutamate decarboxylase (GAD, *ptos_000367*), which converts glutamate to γ-amino butyric acid (GABA), is also activated. This reaction consumes a proton and is thought to contribute to neutralization of an acidified cytoplasm (4). Glutamate decarboxylase is associated with tolerance of acid and bile acid stress in many bacteria, including *Bacteroides* species (5-7) . Most of these genes have neutral fitness scores. However strains with disruptions in *gdhA*, whose activity results in the generation of reducing equivalents, have a fitness advantage. This result is consistent with the fitness advantage of disrupting other pathways that result in the generation of reducing equivalents, namely the oxidative phase of the pentose phosphate pathway (*zwf*, *pgl*, *gnd*), and the first step of beta-oxidation of fatty acids (*fadE* and *fixAB*) (main text Figure 3 & 5).

A second set of upregulated metabolic genes have predicted roles in citrate/carboxylate metabolism (enriched GO term: tricarboxylic acid cycle) including the gene cluster *ptos_003272-3274* (citrate synthase, isocitrate dehydrogenase, and aconitase) and a succinyl-CoA ligase system (*ptos_001896-1897*). Activation of lactate/malate dehydrogenase (*ptos_000443*) and L-lactate permease (*ptos_001319*) expression by approximately 50-fold in the presence of DC indicates a shift in carboxylic acid metabolism in *B. fragilis* during bile stress. Finally, transcription of multiple enzymes comprising the pentose phosphate pathway (enriched GO term: pentose-phosphate shunt) including *gnd*, *zwf*, and *pgl* (*ptos_1536-1538*), *fba* (*ptos_002818*) and *rpiA* (*ptos_003205*) are activated by DC. Other genes in this pathway including *rpiB* (*ptos_001356*) and *ptos_002671* and *ptos_002867* are repressed by DC (Table S1). This pathway produces key substrates for anabolic metabolism and also yields reducing equivalents.

DC-regulated expression of membrane ion transport systems

Transcription of the F_OF_1 ATP synthase operon (*ptos_001821-1829*) is activated upon DC exposure (enriched GO terms: proton motive force-driven ATP synthesis), suggesting that DC compromises energy production in *B. fragilis*, perhaps by affecting membrane permeability. Indeed, expression of multiple genes with predicted roles in gated ion transport (enriched GO terms: monoatomic ion gated channel activity) (Table S2) is activated by DC including ion channels *ptos_003102* and *ptos_000442*, mechanosensitive ion channels *ptos_002809*, *ptos_004068*, *ptos_000516*, *ptos_000659*, *ptos_000815*, and the H(+)/Cl(-) exchange transporter *ptos_001050*. Overall, we observe a variable pattern of activation and repression of annotated ion transporters with the highest level of activation (≈20-fold) for a Na+/H+-translocating pyrophosphatase family protein, *ptos_000210* (8, 9), and transporters with predicted roles in sulfate (*ptos_002300*) and zinc transport (*zup; ptos_002150*). The large effect of DC treatment on ion transport gene transcription provides evidence of general dysregulation of cellular ion homeostasis.

bioRxiv preprint doi: https://doi.org/10.1101/2023.05.11.540287; this version posted October 17, 2023. The copyright holder for this preprint (which was not certified by peer review) is the author/funder, who has granted bioRxiv a license to display the preprint in perpetuity. It is made available under aCC-BY 4.0 International license.

***B. fragilis* P207 relies on multiple efflux and stress response systems in the presence of bile**

Efflux is a well-established mechanism of bile-tolerance (10). TolC-family outer membrane proteins work in conjunction with RND- and ABC- family transport systems to enable efflux of a broad range of substrates, including bile acids (11, 12). These tripartite efflux systems are comprised of an inner membrane permease, a TolC-family protein in the outer membrane, and a periplasmic adapter protein that bridges the inner and outer membrane proteins. *B. fragilis* P207 encodes 23 TolC-family exporter systems, most of which are not strongly expressed in BHIS (Figure S8). Previous work in *B. thetaiotaomicon* identified a TolC-type bile-induced efflux system, BT2792-BT2795, that specifically contributed to fitness in the presence of multiple bile acids (13). *B. fragilis* P207 does not encode an orthologous system, but we identified multiple tripartite TolC-containing efflux systems that contribute to fitness in DC and BEP, likely in a redundant fashion (Figure S7). The overall repertoire of tripartite efflux genes that support growth/survival in DC and BEP is similar, though reduced fitness due to disruption of *ptos_000132-34* is specific to DC.

While both our transcriptomic and functional genomic datasets provide evidence that efflux systems are important in the context of bile, these approaches highlight distinct efflux systems (Figure S7). For example, the TolC-family protein encoded by *ptos_003611* is the most highly induced gene in the DC transcriptomic data set (main text Figure 2, Table S1) yet the fitness consequence of disrupting this gene or adjacent transport genes is minor in DC (Figure S7). We note that *ptos_003611-14* does contribute to fitness in crude BEP raising the possibility that DC may act as a signal to upregulate expression of a system that is important for export of other molecules that often co-occur with DC in the mammalian gut. No single system is critical for survival in DC; instead *ptos_000132-34*, *ptos002878-80* and *ptos_03242-44* each contribute to fitness, likely in a redundant fashion. Transcription of *ptos_003730-32* is activated by DC treatment but insertions in this locus do not impact fitness in our conditions. However, Tn-*Himar* insertions in the adjacent transcriptional repressor gene, *ptos_003733*, confer a fitness advantage in the presence of DC and BEP (Figure S7). From this result, we infer that de-repression of *ptos_003730-32* via disruption of its transcriptional repressor promotes growth and/or survival in DC and BEP. Together these data indicate multiple efflux systems are transcriptionally regulated by and/or contribute to fitness in the presence of bile.

Distinct sets of stress response genes are identified in our transcriptomic and functional genomic experiments. Among the most highly activated genes in the transcriptional dataset are those with known roles in acute stress responses, including genes that mitigate protein misfolding (enriched GO terms: unfolded protein binding; protein folding) (Table S2; main text Figure 2A-B). Genes in this class include the chaperones *groEL*, *groES*, *htpG*, *dnaK*, *clpB*, and a small Hsp20-family protein (*ptos_002388*). Most of these genes are not represented in our fitness data (Table S6, Figure S9). On the other hand, transcripts corresponding to the protein chaperone *dnaJ* (*ptos_001434*), which is important for maintaining proteostasis during stress, are only modestly elevated in DC, but strains with disruptions in this gene are strongly attenuated in all bile conditions. The ClpXP protease system encoded by *ptos_003701-2* also fosters protein quality control during stress. Similar to *dnaJ*, these genes are critical for fitness in bile, but are not transcriptionally regulated by DC exposure (main text Figure 3, Figure S9, Table S1 & S6). Thus both data sets provide evidence that growth in bile involves mitigation of protein misfolding. We observe transcriptional responses indicative of oxidative stress in DC (enriched GO terms: response to oxidative stress; cellular oxidant detoxification; and cellular homeostasis) (Table S2). Specifically, *msrB*, which repairs oxidatively-damaged methionine residues, *katB* catalase (*ptos_001055*), superoxide dismutase (*ptos_002119*), peroxide stress protein YaaA (*ptos_003649*) and glutathione peroxidase (*ptos_003459*) are all strongly activated by DC exposure, as are DNA starvation/stationary phase protection protein (*dps; ptos_001118*), and universal stress protein (*usp*; *ptos_002049*) (Table S1, main text Figure 2A-B). However, none of these genes exhibit fitness defects when disrupted (Figure S9, Table S6). Functional redundancy in these oxidative stress responses likely protects cells from loss of any single gene in this group. We further observed induction of all three genes involved in production of inorganic polyphosphate (*ptos_002410, ptos_002960, ptos_003236*), which is known to confer general protective effects during stress exposure in a variety of bacterial taxa (14-17). Again, disruption of any one of these three polyphosphate kinases is not detrimental in any condition tested (Figure S9, Table S6). Finally, the constitutively expressed *bat* genes (*ptos_02052-58*), which have a reported role in aerotolerance (18), are critical for fitness in our bile conditions (main text Figure 3, Figure S9, Table S6).

Candidate Essential Genes of Note in B. fragilis P207

We note several candidate essential genes that we found to be of particular interest. Transposon insertions were not recovered in one of the two RelA/SpoT paralogs (*ptos_000629*), which regulate levels of the alarmone ppGpp and metabolic adaptation to stress (3). Similarly, in the closely related species *Bacteroides thetaiotaomicron* one of the RelA/SpoT paralogs is also essential (19). Other notable candidate essential genes include a predicted two-gene operon encoding a type III restriction enzyme (*ptos_000974*) and a type III adenine DNA methyltransferase (*ptos_000975*). This gene pair is highly conserved in *B. fragilis* in contrast to most restriction systems, which are often carried on mobile elements and variable among *B. fragilis* isolates. The *rokA* carbohydrate kinase (*ptos_000462*) has been successfully deleted in a derivative of *B. fragilis* strain 638R (20), but is classified as a candidate essential gene in P207 in our growth conditions based on HMM analysis of insertion data. A *upxY* family transcription antiterminator (*ptos_002531*) is classified as essential by HMM. The orthologous gene in *B. fragilis* 638R (BF638R_2798) was also classified as essential in a global Tn-seq study (21). This *upxY* paralog is not

bioRxiv preprint doi: https://doi.org/10.1101/2023.05.11.540287; this version posted October 17, 2023. The copyright holder for this preprint (which was not certified by peer review) is the author/funder, who has granted bioRxiv a license to display the preprint in perpetuity. It is made available under aCC-BY 4.0 International license.

adjacent to a corresponding *upxZ* anti-antiterminator, as is typical in *Bacteroides* capsular polysaccharide loci (22). Rather, *ptos_002531* is the first gene in a putative 28 gene operon that encodes the machinery to synthesize the large capsule EPS elaborated on a fraction of cells in a population (23). Within this large predicted operon, insertion in all downstream genes are recovered, though a gene of unknown function (*ptos_002544*) and a glycosyltransferase family 4 gene (*ptos_002546*) are scored as growth defective based on the distribution of insertions recovered (Table S5).

Supplemental Figures

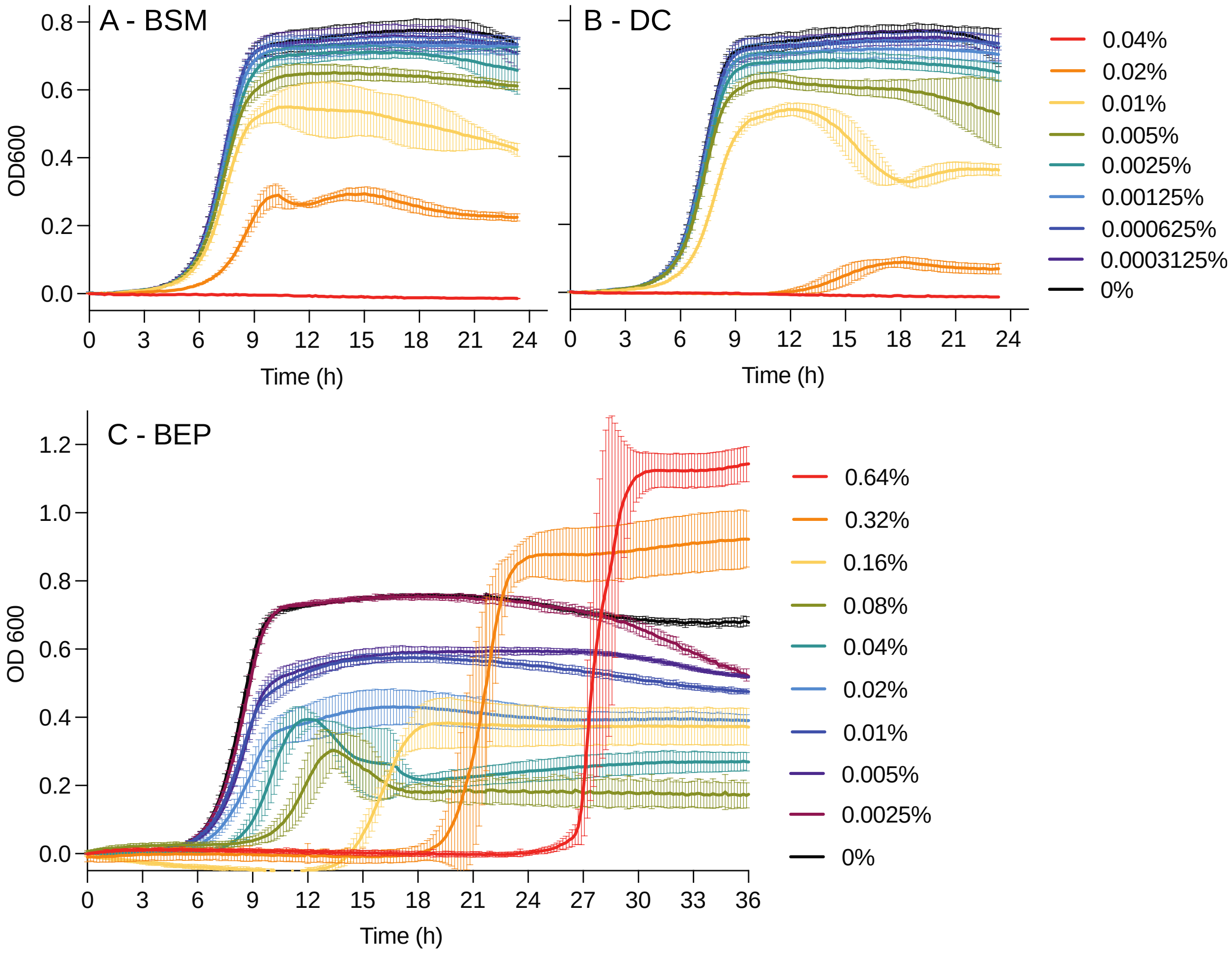

Figure S1: Representative growth curves of *B. fragilis P207* grown in BHIS in the presence of increasing concentrations of bile. A. Bile salt mixture (BSM), **B.** deoxycholate (DC), **C.** bile extract of porcine (BEP). Concentrations are in % w/v. The optical density of 200ul of culture in 96-well plates was measured every 10 minutes. Data represent the mean ± SD of 4 replicate cultures grown in the same 96-well plate. The highest concentrations of BEP delayed growth, but enhanced total culture yield.

bioRxiv preprint doi: https://doi.org/10.1101/2023.05.11.540287; this version posted October 17, 2023. The copyright holder for this preprint (which was not certified by peer review) is the author/funder, who has granted bioRxiv a license to display the preprint in perpetuity. It is made available under aCC-BY 4.0 International license.

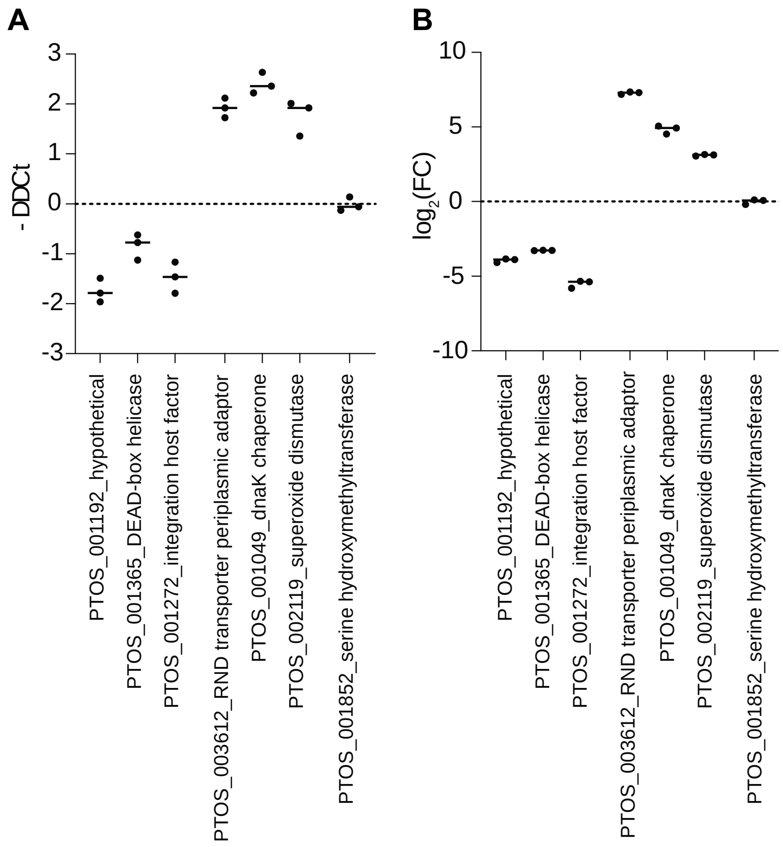

Figure S2: Evaluation of changes in transcript abundance 20 minutes after deoxycholate treatment by RT-qPCR. A. Relative abundance of select transcripts evaluated by RT-qPCR compared to a reference gene (PTOS_001202, Sigma^{70} RNA polymerase sigma factor), where $-\Delta\Delta Ct$ is the calculated as $-((Ct_x-Ct_{ref})_{20\ min}-(Ct_x-Ct_{ref})_{0\ min})$. **B.** Relative abundance of the same set of genes evaluated by RNA seq, where fold change (FC) is average counts $CPM_{0\ min}$/average $CPM_{20\ min}$. The mean and values of triplicate samples for both approaches are plotted. Ct = threshold cycle. CPM = counts per million counts. For three downregulated, three upregulated and one unchanged gene in or RNAseq dataset, the same directionality of change was observed by RT-qPCR. The magnitude of change detected by these two approaches differs. RNAseq is more sensitive to differences and less variable between replicates.

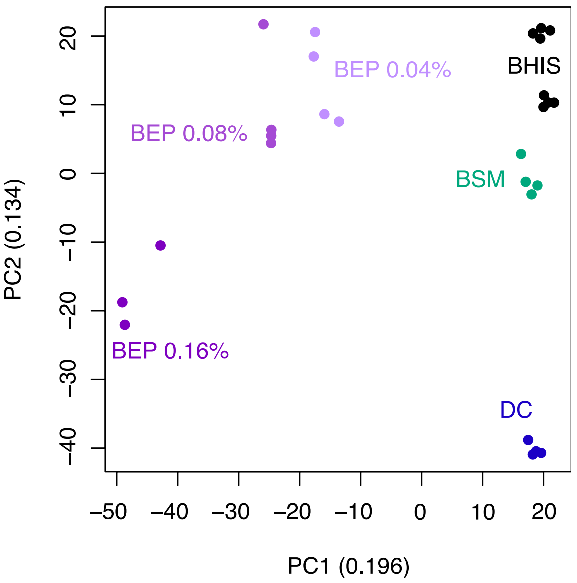

Figure S3: BarSeq replicate data are consistent within each treatment group. Principle component analysis (PCA) of fitness scores from each experimental replicate treatment after the second passage of growth in deoxycholate (DC), bile salt mixture (BSM), plain BHIS, and 0.04%, 0.08%, and 0.16% bile extract porcine (BEP). Genes lacking fitness scores in any experimental set were removed before PCA. The first two principal components are plotted with the fraction of variance accounted for on each axis.

bioRxiv preprint doi: https://doi.org/10.1101/2023.05.11.540287; this version posted October 17, 2023. The copyright holder for this preprint (which was not certified by peer review) is the author/funder, who has granted bioRxiv a license to display the preprint in perpetuity. It is made available under aCC-BY 4.0 International license.

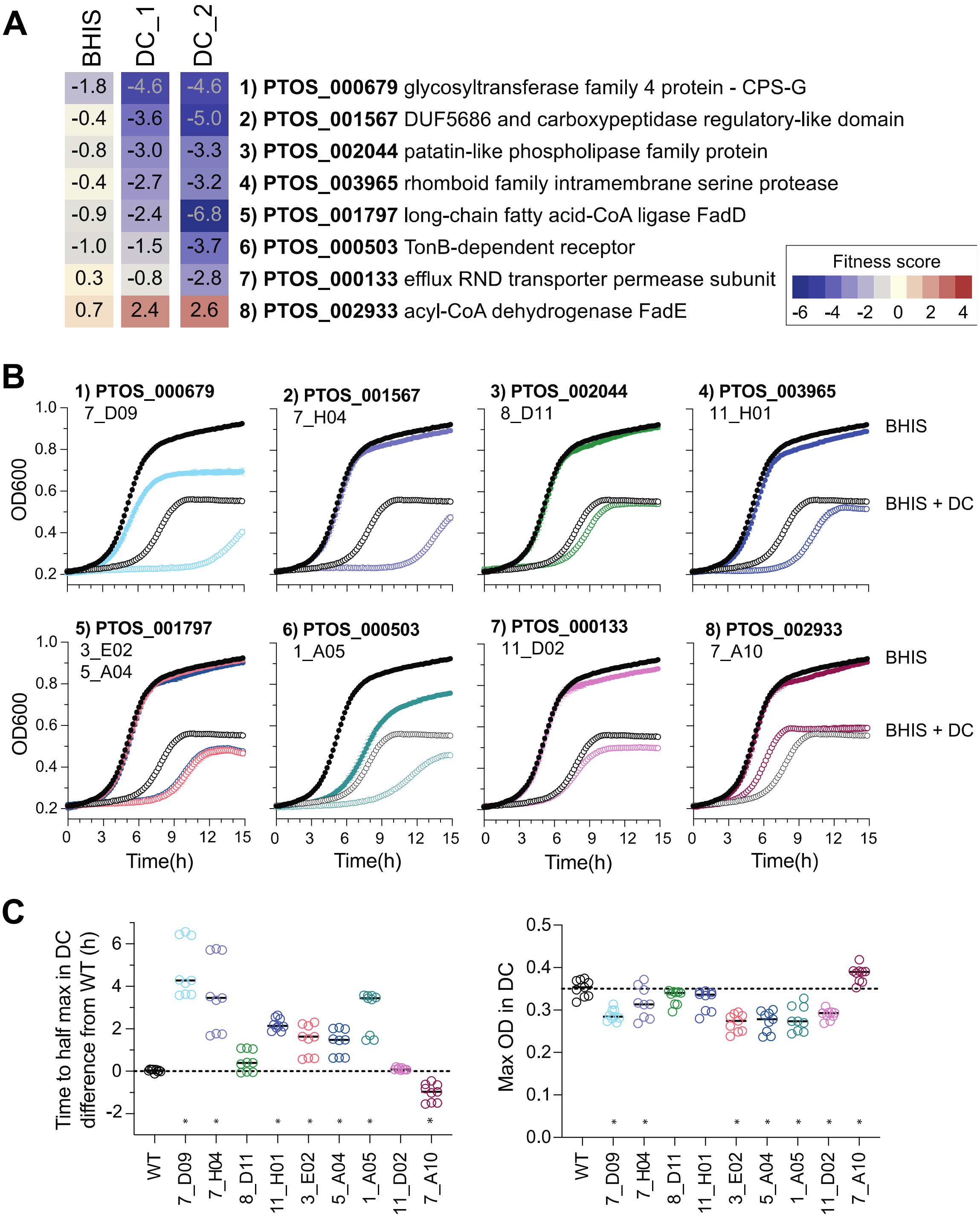

Figure S4: Individual transposon mutant strains validate fitness phenotypes observed in mutant pool. A. Heat map of composite BarSeq fitness scores for 8 genes that were represented by clones in our arrayed collection of mapped transposon mutant strains. Fitness scores after first passage in BHIS or BHIS with DC (DC-1), and also after second passage in BHIS containing DC (DC_2) are indicated. Genes were ranked by fitness score after first passage in DC (DC_1), which most similarly reflects the growth curves below. **B.** Representative growth curves of WT (black circles) and mutant clones (colored circles) grown in BHIS without (closed circles) and with (open circles) 0.01% DC. Growth was monitored by absorbance (OD_{600}) every 10 minutes and data represent mean ± SD of triplicate cultures assayed together. Error bars are often smaller than, and masked by, the symbol. One of three independent trials is presented. The genes and corresponding mutant clone names are indicated in each graph. Our collection contained two strains with insertions in *ptos_001797*, represented by different colors. Most mutants grow similar to WT in the absence of DC, although strains with insertions in *ptos_000679* or *ptos_000503* exhibit defects in BHIS broth alone, consistent with the negative fitness scores in BHIS. **C.** We calculated growth parameters using the Growthcurvr package in R on the growth curves in BHIS+DC on triplicate cultures from three independent trials (n=9). The difference

bioRxiv preprint doi: https://doi.org/10.1101/2023.05.11.540287; this version posted October 17, 2023. The copyright holder for this preprint (which was not certified by peer review) is the author/funder, who has granted bioRxiv a license to display the preprint in perpetuity. It is made available under aCC-BY 4.0 International license.

in time to half-max compared to wild-type ($t\_mid_{mut} – t\_mid_{WT}$), which reflects a composite of differences in lag and growth rate is shown on the left. The max density (k) in BHIS+DC of each growth is shown on the right. In both cases, difference to WT was assessed by one way ANOVA followed by Dunnett's multiple comparisons test (* $p<0.05$). All mutants are statistically different from WT in at least one of these measurements, except 8_D11. The transposon insertion in this strain is in the last 13% of PTOS_002044. It is not uncommon for C-terminal truncations to have modest or no effects. In fact, composite fitness scores ignore strains with insertions in the first or last 10% of a gene because insertions in the termini of gene regions often do not fully disrupt gene function. Overall, the negative fitness scores for genes correspond to either delayed growth and/or lower overall growth in DC for the individual insertion strains compared to WT. Importantly, our collection also contained a strain with an insertion in FadE, which has a positive fitness score in the bulk passaging experiment. The individual strain with an insertion in this gene consistently outperformed WT in the presence of DC, reaching half-max on average an hour sooner than WT and reaching a higher terminal density than WT.

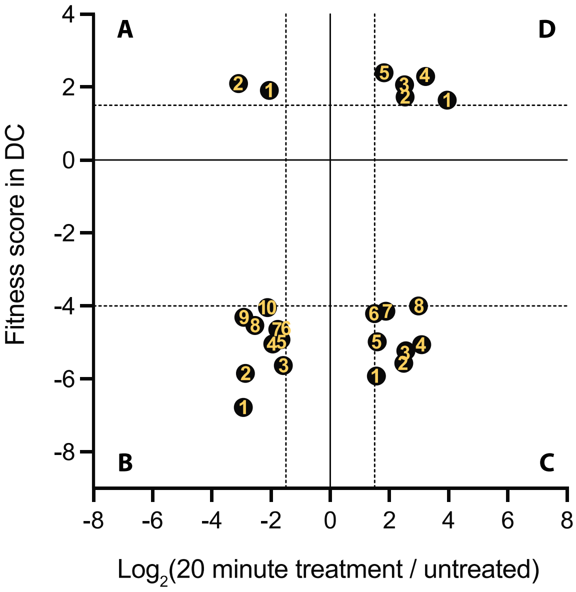

A - Downregulated, beneficial to disrupt
1. PTOS_000763_AIR synthase-related protein
2. PTOS_001773_hypothetical protein

B - Downregulated, detrimental to disrupt
1. PTOS_001797_long-chain fatty acid--CoA ligase
2. PTOS_002991_lipid-A-disaccharide synthase domain-containing
3. PTOS_002992_glycosyltransferase
4. PTOS_001504_GDP-mannose 4,6-dehydratase
5. PTOS_003074_acetyl-CoA carboxylase biotin carboxyl carrier
6. PTOS_003573_TetR/AcrR family transcriptional regulator
7. PTOS_003915_adenosine kinase
8. PTOS_000776_tRNA pseudouridine(38-40) synthase TruA
9. PTOS_002806_hypothetical protein
10. PTOS_004093_cation diffusion facilitator family transporter

D - Upregulated, beneficial to disrupt
1. PTOS_003733_TetR/AcrR family transcriptional regulator
2. PTOS_001537_glucose-6-phosphate dehydrogenase
3. PTOS_000824_hypothetical protein
4. PTOS_001536_phosphogluconate dehydrogenase
5. PTOS_002687_glucosamine-6-phosphate deaminase

C - Upregulated, detrimental to disrupt
1. PTOS_001888_patatin-like phospholipase family protein
2. PTOS_001339_hypothetical protein
3. PTOS_002989_NAD-dependent epimerase/dehydratase
4. PTOS_001191_2-hydroxyacid dehydrogenase
5. PTOS_003802_HDIG domain-containing protein
6. PTOS_002482_V-type ATP synthase subunit K
7. PTOS_003886_helix-turn-helix transcriptional regulator
8. PTOS_003809_aspartate ammonia-lyase

Figure S5: Transcriptionally regulated genes with significant fitness scores. This graph is identical to Figure 4D in the main text, except that the genes in each quadrant (A-D) are numbered. Below the graph are the locus number and predicted annotation corresponding to the numbered genes in each quadrant.

bioRxiv preprint doi: https://doi.org/10.1101/2023.05.11.540287; this version posted October 17, 2023. The copyright holder for this preprint (which was not certified by peer review) is the author/funder, who has granted bioRxiv a license to display the preprint in perpetuity. It is made available under aCC-BY 4.0 International license.

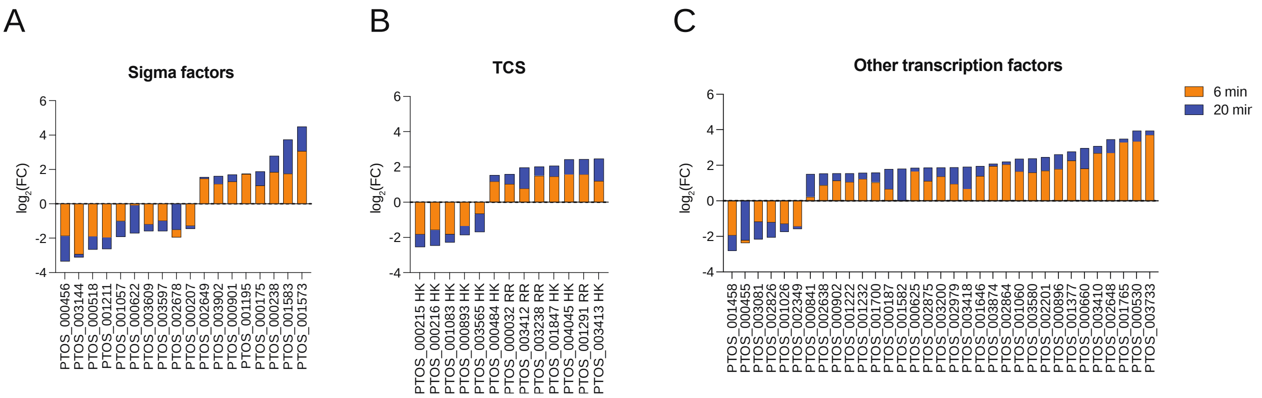

Figure S6: Transcriptional regulators exhibit a complex response to DC. Log2(fold change) in transcript levels of genes encoding **(A)** alternative sigma factors, **(B)** two-component signaling (TCS) proteins (HK – histidine kinase, RR – response regulator), and **(C)** other transcription factors that are themselves transcriptional regulated after 6 or 20 minutes of DC exposure (orange and blue, respectively).

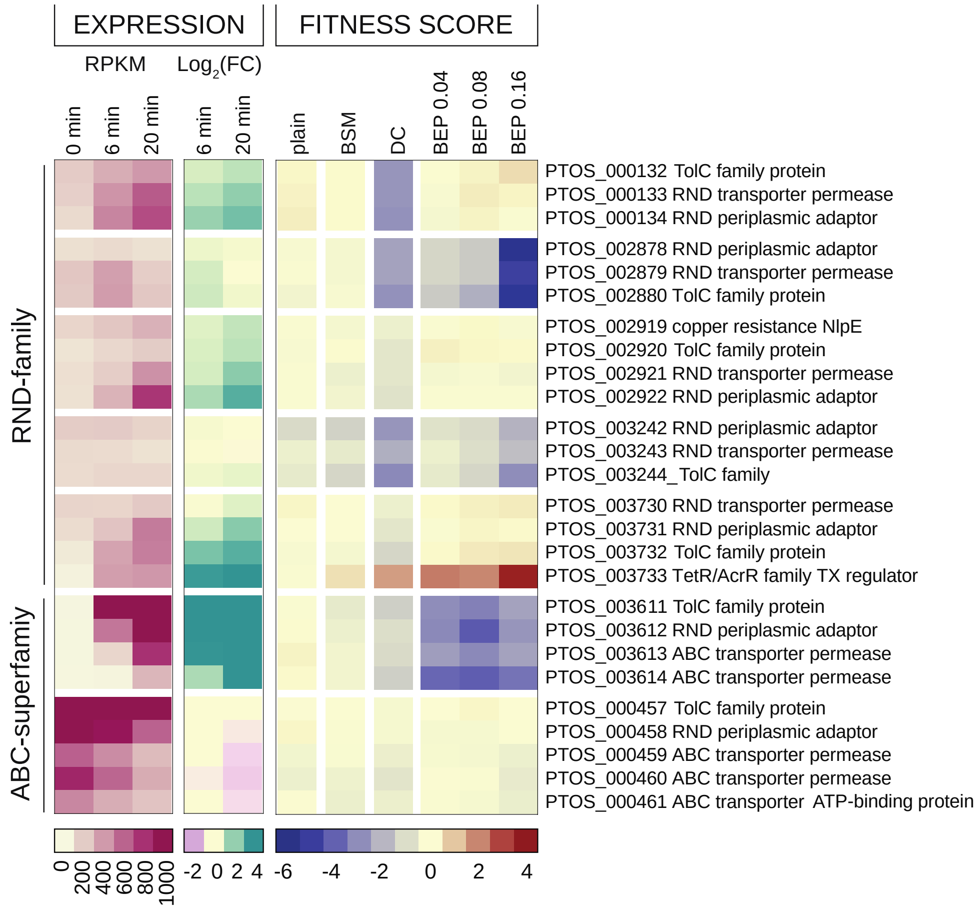

Figure S7: Multiple efflux systems differentially contribute to *B. fragilis* fitness in the presence of purified bile acid and crude bile conditions. Transcriptional and fitness data for TolC-family efflux system that are *either* transcriptionally regulated in the presence of purified deoxycholate *or* contribute to fitness in at least one bile condition (BSM: bile salt mixture, DC: Deoxycholate, BEP: bile extract from porcine). A similar heat map of all 23 TolC-containing operons is presented in Figure S8. Transcript abundance is presented as reads per kilobase per million reads (RPKM) and regulation is indicated by the log2(fold change) compared to untreated cells (0 min). Gene-level fitness scores after the second passage in each of the indicated conditions are presented. Color scales are presented below each type of data.

bioRxiv preprint doi: https://doi.org/10.1101/2023.05.11.540287; this version posted October 17, 2023. The copyright holder for this preprint (which was not certified by peer review) is the author/funder, who has granted bioRxiv a license to display the preprint in perpetuity. It is made available under aCC-BY 4.0 International license.

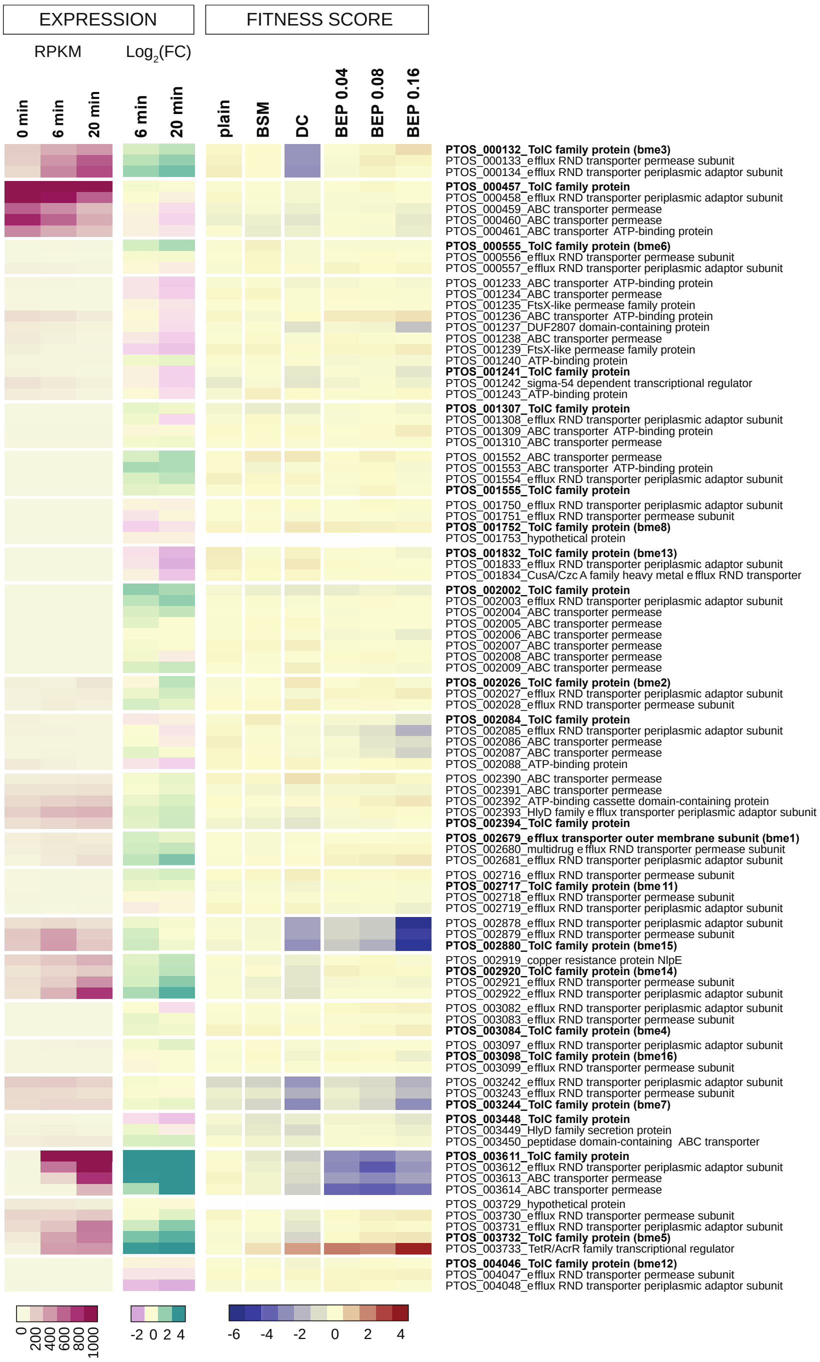

Figure S8: Expression and fitness scores for all 23 TolC-family membrane transport systems. Heat maps transcript levels (RPKM), $\log_2$(fold change) in transcript and gene-level fitness scores after serial passage in the presence of bile (as in Figure S7) for the operons containing each of the 23 TolC-family exporter genes in the *B. fragilis* P207 genome. TolC-family genes are highlighted in bold. Most tripartite efflux systems are not highly expressed in our *in vitro* conditions. Only a small subset contribute to fitness in our bile conditions.

bioRxiv preprint doi: https://doi.org/10.1101/2023.05.11.540287; this version posted October 17, 2023. The copyright holder for this preprint (which was not certified by peer review) is the author/funder, who has granted bioRxiv a license to display the preprint in perpetuity. It is made available under aCC-BY 4.0 International license.

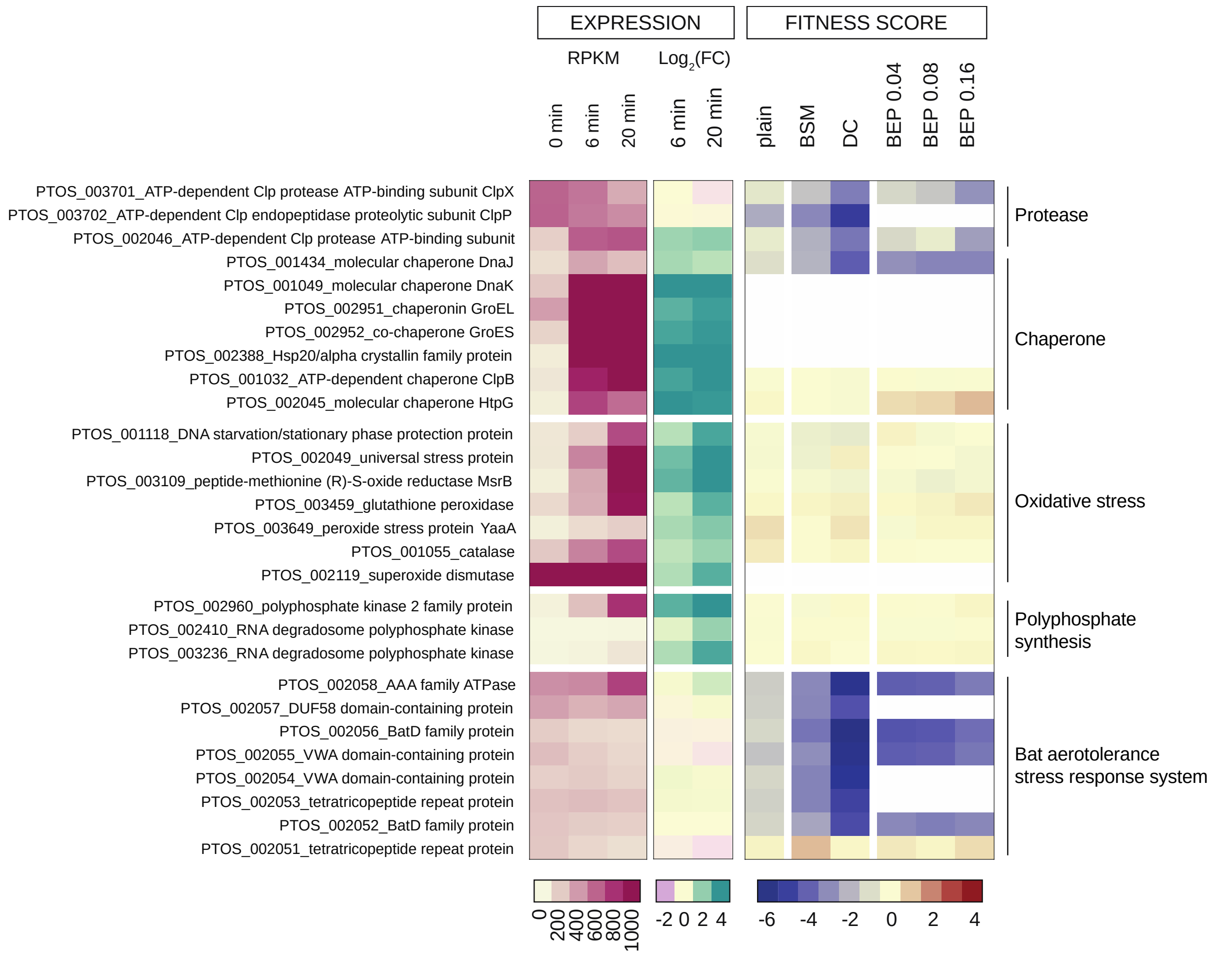

Figure S9: A broad repertoire of stress response systems enable survival in deoxycholate. Heat maps of expression and fitness data for various stress response genes presented as in Figure S7. Protein chaperones, which are important for responding to unfolded proteins or other proteotoxic stress are broadly upregulated in by deoxycholate exposure. The ClpXP protease, which serves a similar function, is not transcriptionally regulated, but is important for fitness. Oxidative stress response genes are broadly upregulated, but no single gene is critical for fitness. Polyphosphate synthesis genes exhibit a similar pattern. The bat system, which has been characterized in aerotolerance, is constitutively expressed and critical for fitness during bile exposure.

bioRxiv preprint doi: https://doi.org/10.1101/2023.05.11.540287; this version posted October 17, 2023. The copyright holder for this preprint (which was not certified by peer review) is the author/funder, who has granted bioRxiv a license to display the preprint in perpetuity. It is made available under aCC-BY 4.0 International license.

Literature cited in supplemental materials

1. Ulrich LE, Koonin EV, Zhulin IB. 2005. One-component systems dominate signal transduction in prokaryotes. Trends Microbiol 13:52-6.
2. Stock AM, Robinson VL, Goudreau PN. 2000. Two-component signal transduction. Annu Rev Biochem 69:183-215.
3. Boutte CC, Crosson S. 2013. Bacterial lifestyle shapes stringent response activation. Trends Microbiol 21:174-80.
4. Feehily C, Karatzas KA. 2013. Role of glutamate metabolism in bacterial responses towards acid and other stresses. J Appl Microbiol 114:11-24.
5. Otaru N, Ye K, Mujezinovic D, Berchtold L, Constancias F, Cornejo FA, Krzystek A, de Wouters T, Braegger C, Lacroix C, Pugin B. 2021. GABA Production by Human Intestinal Bacteroides spp.: Prevalence, Regulation, and Role in Acid Stress Tolerance. Front Microbiol 12:656895.
6. Bron PA, Molenaar D, de Vos WM, Kleerebezem M. 2006. DNA micro-array-based identification of bile-responsive genes in Lactobacillus plantarum. J Appl Microbiol 100:728-38.
7. Begley M, Gahan CG, Hill C. 2002. Bile stress response in Listeria monocytogenes LO28: adaptation, cross-protection, and identification of genetic loci involved in bile resistance. Appl Environ Microbiol 68:6005-12.
8. Luoto HH, Baykov AA, Lahti R, Malinen AM. 2013. Membrane-integral pyrophosphatase subfamily capable of translocating both Na+ and H+. Proc Natl Acad Sci U S A 110:1255-60.
9. Luoto HH, Nordbo E, Baykov AA, Lahti R, Malinen AM. 2013. Membrane Na+-pyrophosphatases can transport protons at low sodium concentrations. J Biol Chem 288:35489-99.
10. Begley M, Gahan CGM, Hill C. 2005. The interaction between bacteria and bile. FEMS Microbiology Reviews 29:625-651.
11. Du D, Wang-Kan X, Neuberger A, van Veen HW, Pos KM, Piddock LJV, Luisi BF. 2018. Multidrug efflux pumps: structure, function and regulation. Nat Rev Microbiol 16:523-539.
12. Anes J, McCusker MP, Fanning S, Martins M. 2015. The ins and outs of RND efflux pumps in Escherichia coli. Front Microbiol 6:587.
13. Liu H, Shiver AL, Price MN, Carlson HK, Trotter VV, Chen Y, Escalante V, Ray J, Hern KE, Petzold CJ, Turnbaugh PJ, Huang KC, Arkin AP, Deutschbauer AM. 2021. Functional genetics of human gut commensal Bacteroides thetaiotaomicron reveals metabolic requirements for growth across environments. Cell Rep 34:108789.
14. Alcantara C, Blasco A, Zuniga M, Monedero V. 2014. Accumulation of polyphosphate in Lactobacillus spp. and its involvement in stress resistance. Appl Environ Microbiol 80:1650-9.
15. Gray MJ, Wholey WY, Wagner NO, Cremers CM, Mueller-Schickert A, Hock NT, Krieger AG, Smith EM, Bender RA, Bardwell JC, Jakob U. 2014. Polyphosphate is a primordial chaperone. Mol Cell 53:689-99.
16. Henry JT, Crosson S. 2013. Chromosome replication and segregation govern the biogenesis and inheritance of inorganic polyphosphate granules. Mol Biol Cell 24:3177-86.
17. Rao NN, Gomez-Garcia MR, Kornberg A. 2009. Inorganic polyphosphate: essential for growth and survival. Annu Rev Biochem 78:605-47.
18. Tang YP, Dallas MM, Malamy MH. 1999. Characterization of the Batl (Bacteroides aerotolerance) operon in Bacteroides fragilis: isolation of a B. fragilis mutant with reduced aerotolerance and impaired growth in in vivo model systems. Mol Microbiol 32:139-49.
19. Schofield WB, Zimmermann-Kogadeeva M, Zimmermann M, Barry NA, Goodman AL. 2018. The Stringent Response Determines the Ability of a Commensal Bacterium to Survive Starvation and to Persist in the Gut. Cell Host Microbe 24:120-132 e6.
20. Brigham CJ, Malamy MH. 2005. Characterization of the RokA and HexA broad-substrate-specificity hexokinases from Bacteroides fragilis and their role in hexose and N-acetylglucosamine utilization. J Bacteriol 187:890-901.
21. Veeranagouda Y, Husain F, Tenorio EL, Wexler HM. 2014. Identification of genes required for the survival of B. fragilis using massive parallel sequencing of a saturated transposon mutant library. BMC Genomics 15:429.
22. Chatzidaki-Livanis M, Weinacht KG, Comstock LE. 2010. Trans locus inhibitors limit concomitant polysaccharide synthesis in the human gut symbiont Bacteroides fragilis. Proc Natl Acad Sci U S A 107:11976-80.
23. Chatzidaki-Livanis M, Coyne MJ, Roche-Hakansson H, Comstock LE. 2008. Expression of a uniquely regulated extracellular polysaccharide confers a large-capsule phenotype to Bacteroides fragilis. J Bacteriol 190:1020-6.
