# Supplementary material for: Multi-omics analysis of a Bacteroides fragilis isolate from an ulcerative colitis patient defines genetic determinants of fitness in bile
Source: bioRxiv. 2023 May 11:2023.05.11.540287. Preprint. [Version 1] doi: 10.1101/2023.05.11.540287 (PMC10197588; doi:10.1101/2023.05.11.540287)
Supplement: Supplementary file 1 [file NIHPP2023.05.11.540287V1-supplement-1.pdf]

bioRxiv preprint doi: https://doi.org/10.1101/2023.05.11.540287; this version posted May 11, 2023. The copyright holder for this preprint (which was not certified by peer review) is the author/funder, who has granted bioRxiv a license to display the preprint in perpetuity. It is made available under aCC-BY 4.0 International license.

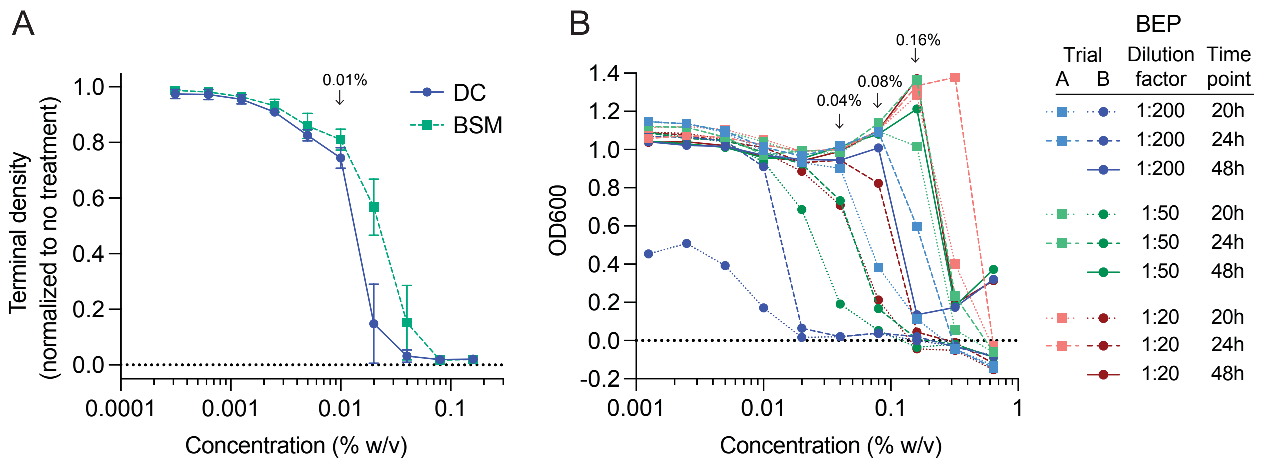

Figure S1: *B. fragilis* P207 growth in the in the presence of increasing concentrations (**A**) deoxycholate (DC) or bile salt mixture (BSM) and (**B**) bile extract of porcine (BEP). Data in A reflect the terminal culture density (OD_{600}) after 24 hours growth; mean ± SD of 4 independent trials each normalized to an untreated control. Data in B represent the culture densities observed in 2 representative trials with different initial inocula (dilution factors from overnight culture) and different time points to show the variability in outcomes. Although cultures with increasing concentrations of BEP grow slower, they reached higher terminal densities consistent with utilization of a component of crude bile for growth.

bioRxiv preprint doi: https://doi.org/10.1101/2023.05.11.540287; this version posted May 11, 2023. The copyright holder for this preprint (which was not certified by peer review) is the author/funder, who has granted bioRxiv a license to display the preprint in perpetuity. It is made available under aCC-BY 4.0 International license.

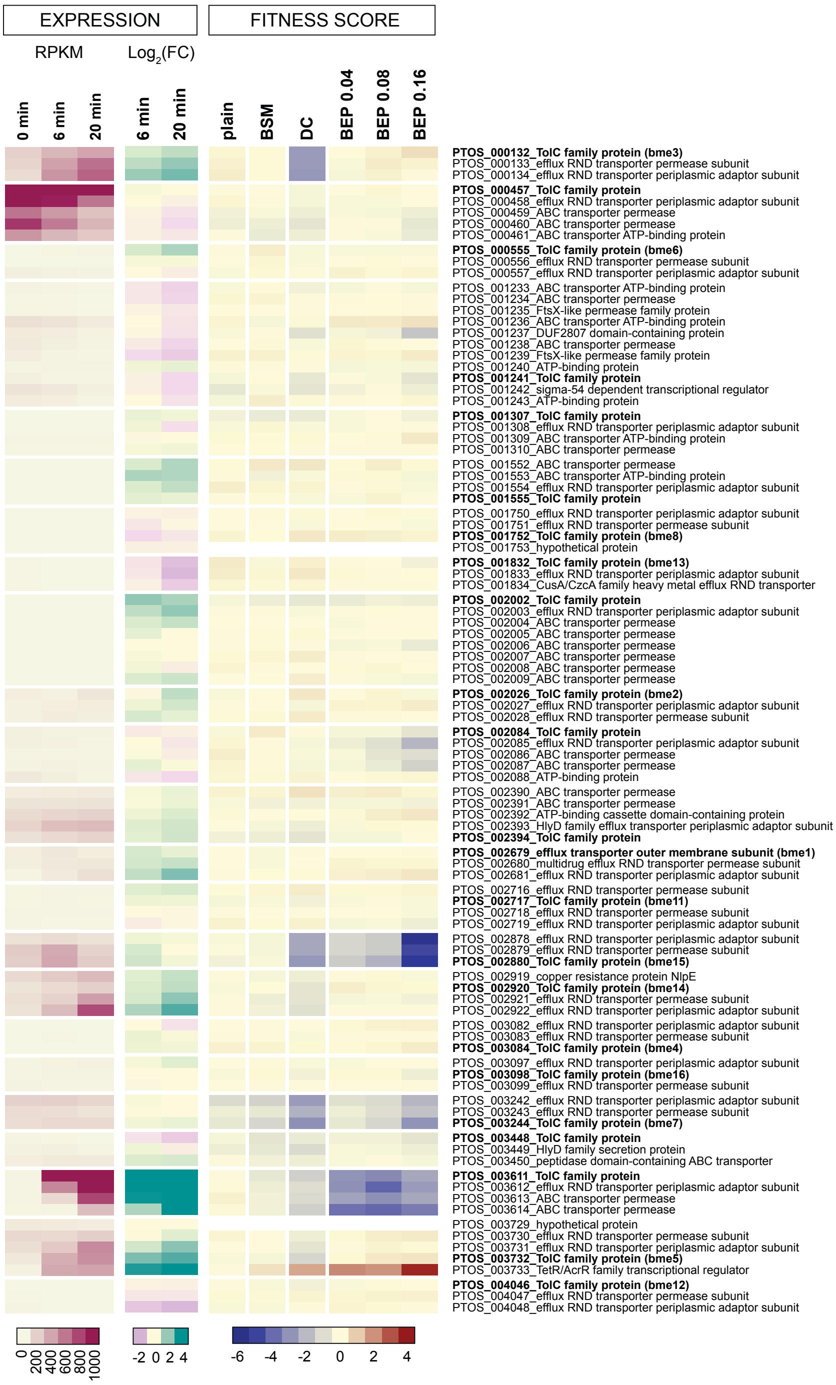

Figure S2: Expression and fitness scores for all 23 TolC-family membrane transport systems. Heat maps of absolute transcript levels (RPKM), $\log_2$(fold change) in transcript levels at 6 minute and 20 minute timepoints after DC treatment (relative to untreated control), and gene-level fitness scores after serial passage in the presence of bile (as in Figure 2) for the operons containing each of the 23 TolC-family exporter genes in the *B. fragilis* P207 genome. TolC-family genes are highlighted in bold. *B. fragilis* RND-TolC systems previously described are indicated in parentheses (64). Color scales representing absolute expression (RPKM), fold change in expression, and mutant fitness scores are presented below each type of data.

bioRxiv preprint doi: https://doi.org/10.1101/2023.05.11.540287; this version posted May 11, 2023. The copyright holder for this preprint (which was not certified by peer review) is the author/funder, who has granted bioRxiv a license to display the preprint in perpetuity. It is made available under aCC-BY 4.0 International license.

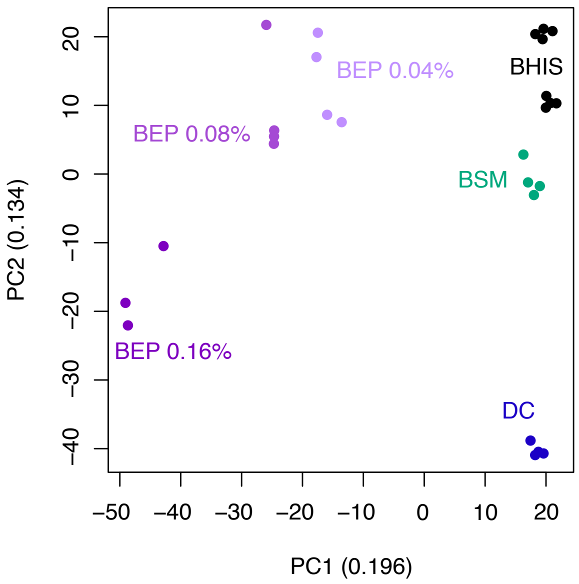

Figure S3: BarSeq replicate data are consistent within each treatment group. Principle component analysis (PCA) of fitness scores from each experimental replicate treatment after the second passage of growth in deoxycholate (DC), bile salt mixture (BSM), plain BHIS, and 0.04%, 0.08%, and 0.16% bile extract porcine (BEP). Genes lacking fitness scores in any experimental set were removed before PCA. The first two principal components are plotted with the fraction of variance accounted for on each axis.

bioRxiv preprint doi: https://doi.org/10.1101/2023.05.11.540287; this version posted May 11, 2023. The copyright holder for this preprint (which was not certified by peer review) is the author/funder, who has granted bioRxiv a license to display the preprint in perpetuity. It is made available under aCC-BY 4.0 International license.

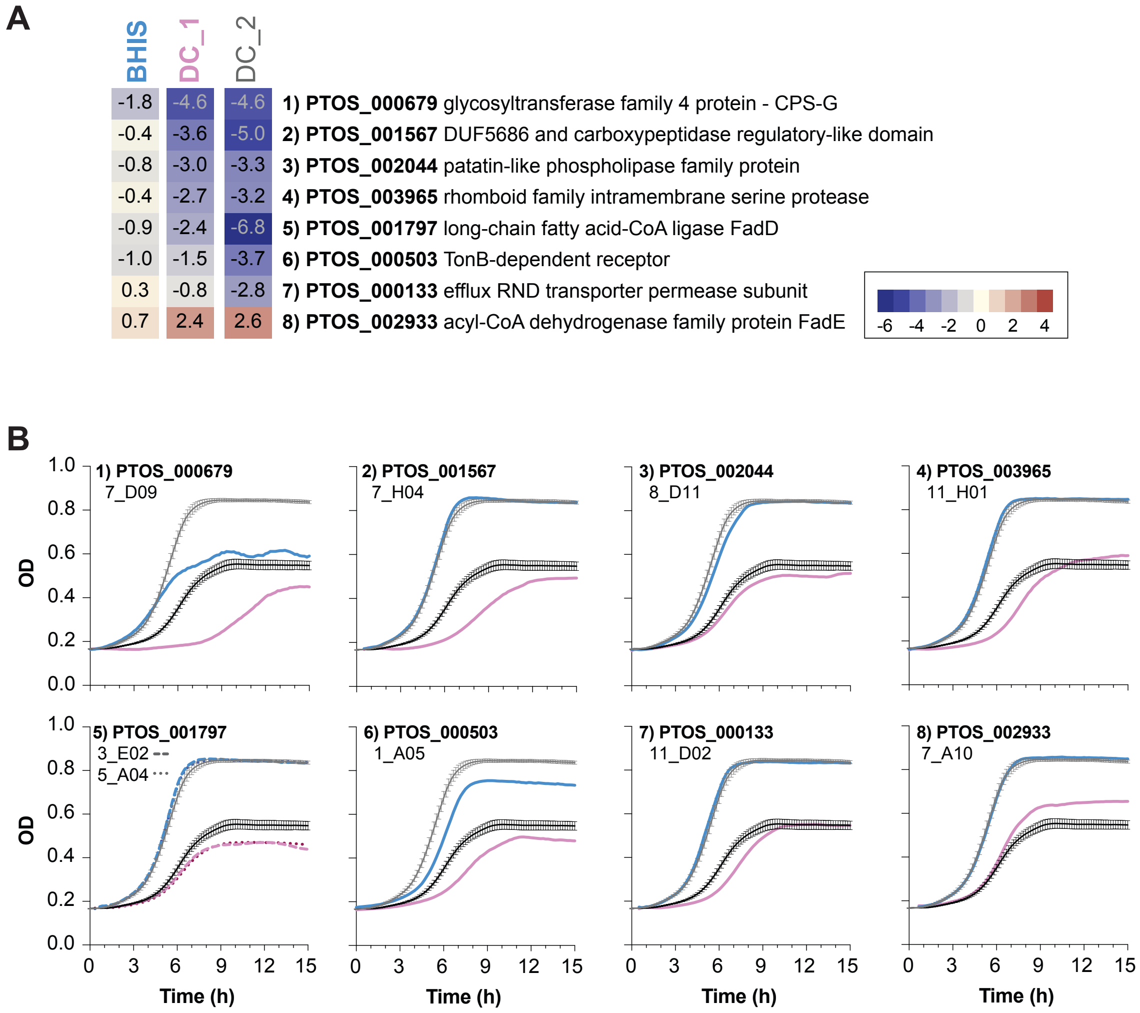

Figure S4: Individual transposon mutant strains validate fitness phenotypes observed in mutant pool. A. Heat map of composite BarSeq fitness scores for 8 genes that were represented by clones in an arrayed mapped collection of transposon mutant strains. Fitness scores after first passage without and with DC (BHIS and DC_1, indicated by blue and pink labels, respectively) and also after second passage in DC (DC_2) are indicated. Genes were ranked by fitness score after first passage in DC (DC_1). **B.** WT and mutant clones were grown in BHIS with and without 0.01% DC in a 96-well plate and monitored by absorbance (OD_{600}). This growth is similar to a single passage of the mutant pool. In each graph, WT growth with and without DC are represented by dark grey and light grey curves, respectively. The genes and corresponding mutant clone names are indicated in each graph. Growth of the mutant strains with and without DC are represented by pink and blue curves, respectively. Our collection contained two strains with insertions in *ptos_001797*, represented by dashed or dotted lines. Most mutants grow similar to WT in the absence of DC, although strains with insertions in ptos_000679 or ptos_000503 exhibit defects in BHIS broth alone, similar to the negative fitness scores in BHIS. The mutants in genes that have negative fitness scores in DC display defects in growth and/or terminal density when cultured in the presence of DC. Importantly, our collection also contained a strain with an insertion in FadE, which has a positive fitness score in the bulk passaging experiment. The individual strain with an insertion in this gene also outperformed WT in the presence of DC.
