# Supplementary material for: Multi-omics analysis of a Bacteroides fragilis isolate from an ulcerative colitis patient defines genetic determinants of fitness in bile
Source: bioRxiv. 2023 May 11:2023.05.11.540287. Preprint. [Version 1] doi: 10.1101/2023.05.11.540287 (PMC10197588; doi:10.1101/2023.05.11.540287)
Supplement: Supplementary file 2 [file media-3.docx]

**Table S3:** ***B. fragilis*** **P207 Tn insertion site mapping statistics, and association of random barcodes with these insertion sites.**

| **Category** | **Value** |
| --- | --- |
| Mapped reads | 10,834,060 |
| Barcodes seen 8 or more times | 82,357 |
| Barcodes mapped reliably (minFrac 0.7 minRatio 7)^a^ | 51,383 |
| Barcodes remaining after masking off-by-1 barcodes | 49,543 |
| Distinct locations hit on B. fragilis chromosome | 43,295 |
| Fraction of reads for those barcodes | 76.6% |
| Insertions in central 10-90% of genes | 37,119 (32310 distinct locations) |
| Protein coding genes with central insertions | 3,489 of 3,967 (87.9%) |
| Strains per hit protein | median 8, mean 10.5 |
| Gene and transposon on same strand | 50.1% |
| Reads per hit protein | median 846, mean 1,456 |
| Reads per million for hit proteins | median 78.1, mean 134.4 |

^a^ minFrac is the minimum fraction of reads for the barcode that agree with the preferred mapping . minRatio is the minimum ratio of reads for preferred mapping over the 2nd-most-frequent mapping based on code available at https://bitbucket.org/berkeleylab/feba/src/master/
